# Mitochondrial ATP Production is Required for Endothelial Cell Control of Vascular Tone

**DOI:** 10.1093/function/zqac063

**Published:** 2022-12-09

**Authors:** Calum Wilson, Matthew D Lee, Charlotte Buckley, Xun Zhang, John G McCarron

**Affiliations:** Strathclyde Institute of Pharmacy and Biomedical Sciences, University of Strathclyde, 161 Cathedral Street, Glasgow G4 0RE, UK; Strathclyde Institute of Pharmacy and Biomedical Sciences, University of Strathclyde, 161 Cathedral Street, Glasgow G4 0RE, UK; Strathclyde Institute of Pharmacy and Biomedical Sciences, University of Strathclyde, 161 Cathedral Street, Glasgow G4 0RE, UK; Strathclyde Institute of Pharmacy and Biomedical Sciences, University of Strathclyde, 161 Cathedral Street, Glasgow G4 0RE, UK; Strathclyde Institute of Pharmacy and Biomedical Sciences, University of Strathclyde, 161 Cathedral Street, Glasgow G4 0RE, UK

**Keywords:** endothelial cell, mitochondria, ATP, vasodilation, blood flow

## Abstract

Arteries and veins are lined by nonproliferating endothelial cells that play a critical role in regulating blood flow. Endothelial cells also regulate tissue perfusion, metabolite exchange, and thrombosis. It is thought that endothelial cells rely on ATP generated via glycolysis, rather than mitochondrial oxidative phosphorylation, to fuel each of these energy-demanding processes. However, endothelial metabolism has mainly been studied in the context of proliferative cells, and little is known about energy production in endothelial cells within the fully formed vascular wall. Using intact arteries isolated from rats and mice, we show that inhibiting mitochondrial respiration disrupts endothelial control of vascular tone. Basal, mechanically activated, and agonist-evoked calcium activity in intact artery endothelial cells are each prevented by inhibiting mitochondrial ATP synthesis. Agonist-evoked calcium activity was also inhibited by blocking the transport of pyruvate, the master fuel for mitochondrial energy production, through the mitochondrial pyruvate carrier. The role for mitochondria in endothelial cell energy production is independent of species, sex, or vascular bed. These data show that a mitochondrial ATP supply is necessary for calcium-dependent, nitric oxide-mediated endothelial control of vascular tone, and identifies the critical role of endothelial mitochondrial energy production in fueling perfused blood vessel function.

## Graphical Abstract

**Graphical Abstract:**
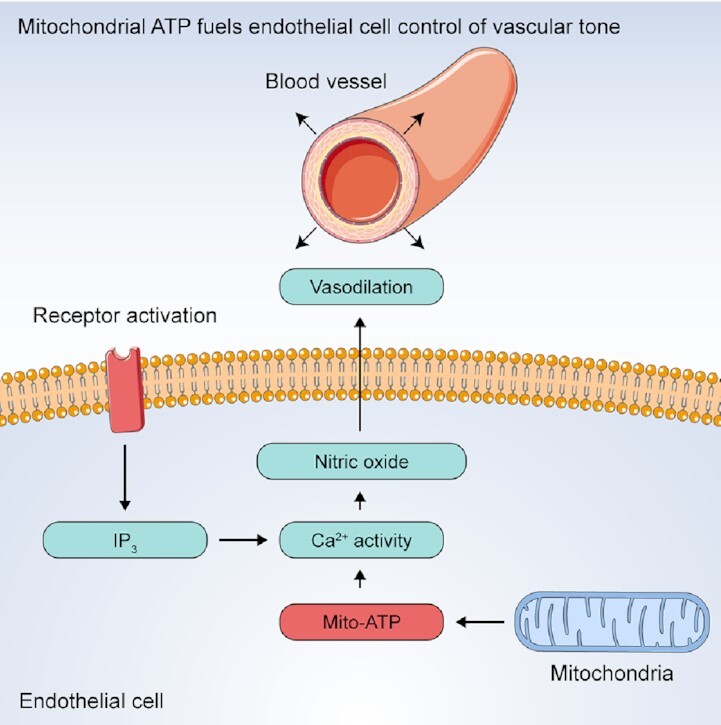

## Introduction

Endothelial cells (ECs) are one of the most abundant mammalian, non-blood cells in the body,^1^,^2^ and they form the inner lining of all blood vessels. In most adult tissue, they exist in a state characterized by minimal or absent migration and proliferation. Indeed, human coronary ECs in vivo have a slow replication rate, with renewal of the entire population taking approximately 6 years.^3^ Whilst EC replication in mature blood vessels is negligible, these cells are highly active and play a crucial role in controlling blood flow by regulating blood vessel contraction and dilation. ECs also regulate the movement of fluid, metabolites, and other cells between the bloodstream and body tissue. Impairment of such EC functions precipitates, aggravates, and reinforces cardiovascular diseases such as atherosclerosis and hypertension.

Alongside negligible replication rates, ECs in perfused blood vessels are usually considered to have a low basal metabolism.^4^ Nevertheless, endothelial function requires energy. In most cells, the bulk of energy is provided by mitochondria in the form of ATP. But under oxygen-limiting conditions, cells are forced to rely on glycolysis for ATP production. Remarkably, despite ECs having abundant access to high concentrations of oxygen in the blood, four decades of reports have indicated that they use glycolysis for ATP production rather than mitochondrial oxidative phosphorylation.^5–8^ The preference for glycolysis is proposed to allow ATP to be generated: (1) at a faster rate; (2) in the absence of oxygen; (3) to facilitate oxygen transfer to extravascular cells; and (4) to protect ECs from reactive oxygen species (ROS). Together with observations that mitochondria occupy a smaller fraction of the volume of ECs than the organelles do in more energetic cells such as hepatocytes and cardiac myocytes (∼5% vs. ∼30%),^9^,^10^ these proposals are used to justify the assumption that mitochondrial respiration is dispensable for endothelial physiology.^11–15^

Recent findings have placed considerable emphasis on this prevailing, but potentially incomplete, view of mitochondria as a minor source of ATP production in ECs. This is particularly true for the study of angiogenesis, where a number of important studies have highlighted how EC glycolysis drives blood vessel growth (see, eg, among a deluge of review articles those by Falkenberg et al. (2019) and Li et al. (2019)^16^,^17^). But this metabolism–function relationship has also led to a reappraisal of the role of mitochondrial energy production in EC function.^18^ For example, new findings have revealed that global pharmacological inhibition and conditional, endothelial-specific knockout of the mitochondrial respiratory chain complex III each inhibit endothelial-driven wound healing.^19^,^20^ Other studies demonstrate that mitochondrial respiration regulates fatty acid transport across the vascular wall,^21^ and is necessary for angiogenesis.^22–25^ Together, these studies suggest that endothelial mitochondria do play an important role in vascular function. However, the significance of mitochondrial metabolism has rarely been studied outside of the context of proliferative ECs in angiogenesis or pulmonary hypertension,^26^ in which the contribution of the organelle may be diminished. Moreover, almost no attention has been given to the energetic requirements of perfused blood vessel ECs.

In the present study, we tested the necessity of mitochondrial ATP production for the obligatory role of ECs in the relaxation of arterial smooth muscle.^27^ We found that preventing EC production of ATP at mitochondrial complex V increases blood vessel contraction and inhibits endothelium-dependent blood vessel relaxation. The requirement for mitochondrial ATP production is independent of sex, species, or vascular bed. Our results provide evidence that mitochondrial energy production in ECs critically regulates blood vessel diameter. Moreover, mitochondrial energy production is far more intimately linked to EC physiology than is currently appreciated.

## Results

### Mitochondrial ATP Fuels Endothelial Nitric Oxide-Mediated Control of Vascular Tone

**Figure 1.**
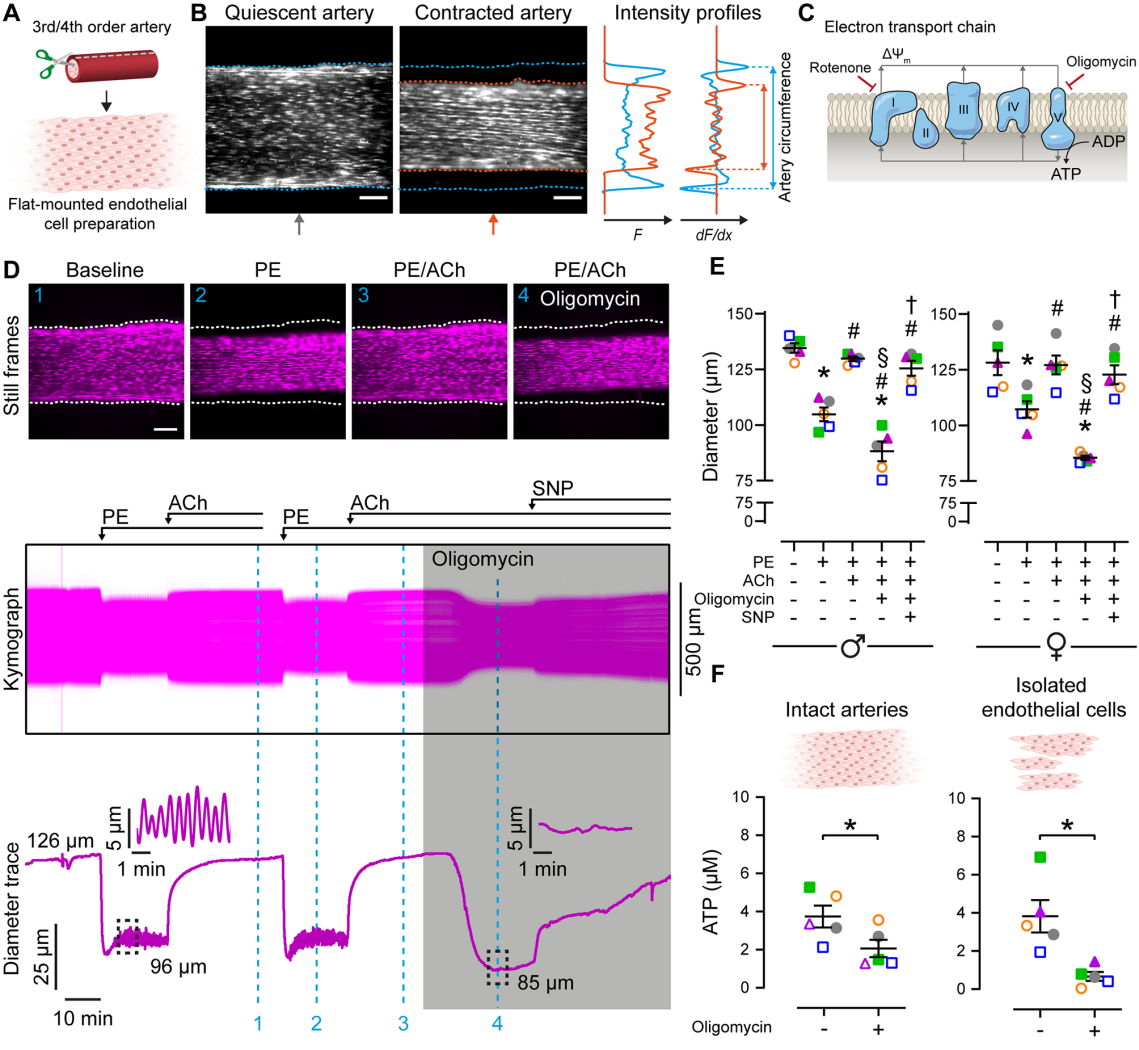
Inhibiting oxidative phosphorylation reverses endothelium-dependent vasodilation. (A) Schematic of dissection procedure for en face EC imaging of flat-mounted blood vessels. Blood vessels are cut open and mounted in a low-volume imaging chamber with the endothelium facing up. Agents that alter vascular activity are delivered using a continuous perfusion system. (B) Still frame images of mesenteric artery ECs before and during stimulation of smooth muscle cell contraction. The position of the artery edges is determined using the derivative of intensity profiles that bisect the artery. (C) Scheme depicting the sites of action for mitochondrial inhibitors used in the study. (D) Still frame images, kymograph, and calculated diameter trace showing the effect of oligomycin (2.4 μM) on repeatable vasodilation to ACh (10 μM) in arteries contracted with phenylephrine (PE, concentration adjusted to achieve ∼20% contraction). Sodium nitroprusside (SNP; 100 μM) was used to test endothelium-independent vasodilation. Data shown in Video 1. (E) Summary data (mean ± SEM overlaid) showing the effect of oligomycin on vasomotor responses in mesenteric arteries from male and female rats. Each color-coded set of data points represents repeat measurement from a single artery (each from a different animal, *n* = 5 in each experiment). The dataset in (D) is from a female rat and is shown in (E) as magenta triangles. (F) Summary data showing cellular ATP in intact mesenteric microvessels and in freshly isolated ECs. Significance markers indicates statistical significance (*P* < .05) using repeated measures one-way ANOVA with Tukey’s test for multiple comparisons (* vs. baseline; # vs. PE; § vs. PE/ACh; † vs. PE/ACh/oligomycin) or paired *t-*test (F). Image scale bars = 100 μm.

To investigate the role of mitochondrial energy metabolism in endothelial nitric oxide-mediated blood flow control, we used a single-photon microscope optimized to visualize ECs and detect increases or decreases in vascular tone in small mesenteric arteries (Figure 1A and B).^28^,^29^ The α-adrenergic receptor agonist, phenylephrine (PE), was used to constrict arteries and either the muscarinic receptor agonist, acetylcholine (ACh) or the selective TRPV4 activator, GSK1016790A, to elicit blood vessel relaxation. ACh was chosen as it selectively activates nitric oxide-mediated endothelium-dependent vasodilation in mesenteric arteries (Figure S1A–C). We selected GSK1016790A for two main reasons: (1) TRPV4 channels activate vasodilation via the endothelium-dependent hyperpolarization pathway in rat mesenteric arteries (Figure S1D–F)^28^; and (2) TRPV4 channel activity persists in the absence of intracellular ATP.^30^,^31^

First, vessel tone was monitored as arteries were stimulated with PE and then ACh in the absence and presence of the mitochondrial ATP synthase (complex V) inhibitor, oligomycin (2.4 µM; Figure 1C and D, Video 1). The concentration of PE (∼2 µM) was titrated to achieve a ∼20% increase in artery tone in control conditions. At the plateau phase of the constriction, ACh (10 µM) evoked a robust dilation. Following washout, vasomotor responses were reproducible. Significantly, subsequent inhibition of mitochondrial ATP production using oligomycin reversed relaxations to ACh. The reversal of ACh-evoked dilation by oligomycin occurred within 10 min and was observed in arteries from both male and female rats (Figure 1E). However, oligomycin did not prevent endothelium-independent vasodilation to sodium nitroprusside (SNP, 100 µM; Figure 1D and E). In contrast, TRPV4-mediated vasodilation persisted after we inhibited mitochondrial ATP production with oligomycin (Figure S1G and H). Importantly, and consistent with its role as an inhibitor of the ATP synthase, we found that oligomycin (2.4 µM)-depleted cellular ATP levels in intact blood vessels and in freshly isolated sheets of ECs (Figure 1F). Moreover, the functional impairment induced by mitochondrial complex V inhibition was mimicked by rotenone (500 nM), a known inhibitor of mitochondrial respiration at complex I (Figure S2A and B). Collectively, these data suggest that mitochondrial ATP modulates nitric oxide-mediated control of vascular tone.

To examine whether mitochondrial ATP depletion prevented EC vasodilator signaling or induced smooth muscle cell contraction, or both, we preincubated arteries with oligomycin and re-examined vascular reactivity. When applied under resting conditions (in the absence of vasoactive stimuli) oligomycin did not evoke vascular contraction. However, subsequently, PE-induced artery constriction was enhanced and ACh-evoked dilation was impaired (Figure S3A and B). Again, oligomycin did not prevent endothelium-independent relaxations to SNP (Figure S3B). The effects of oligomycin (increased vessel constriction, decreased dilation) mimic those induced by mechanical ablation of the EC layer.^28^ The only known non-mitochondrial target of oligomycin, the plasmalemmal Na^+^/K^+^-ATPase,^32^ was not responsible for these effects since ouabain (10 µM), the specific inhibitor of this enzyme, did not prevent ACh-evoked vasodilation (Figure S3C). In addition, removal of glucose from the perfusate had no effect on ACh-evoked vasodilation suggesting that glycolysis plays a minor role in endothelial vasomotor control (Figure S3D). These data confirm that ECs are the site of action of the mitochondrial inhibitors.

**Figure 2.**
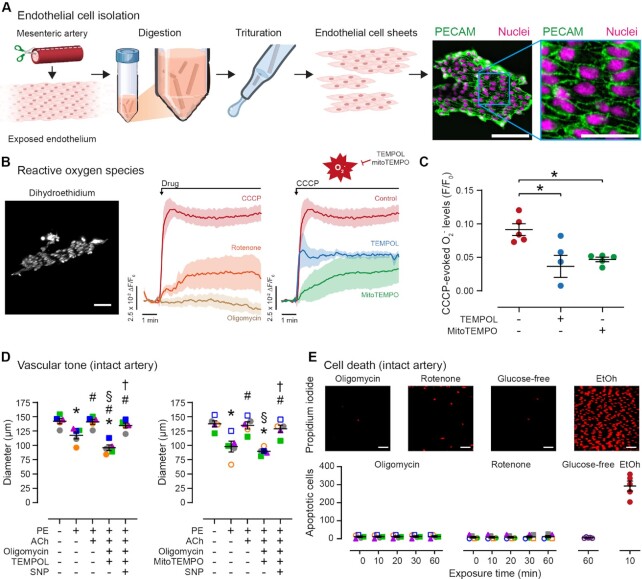
Oligomycin does not promote superoxide production or cell death in native ECs. (A) Schematic of EC isolation procedure and fluorescence image of an isolated EC sheet stained with the nuclear stain, DAPI, and for the endothelial specific marker, platelet EC adhesion molecule (PECAM). Scale bar = 50 μm (25 μm, inset). (B) and (C) Still frame images (scale bar = 50 μm), mean ± SEM time courses, and summary data showing: (1) the effect of mitochondrial inhibitors (CCCP, 5 μM; rotenone, 500 nM; oligomycin, 2.4 μM) on endothelial superoxide levels; and (2) the effect of global (TEMPO, 100 μM) or mitochondrial-targeted (mitoTEMPOL, 50 μM) ROS scavengers on CCCP-evoked increases in EC superoxide levels (*n* ≥ 4). (D) Summary data showing the effect of ROS scavenging on the reversal of ACh-evoked (10 μM) vasodilation following mitochondrial ATP synthase impairment (using oligomycin; 2.4 μM). (E) Example images (scale bars = 50 μm) and summary data showing the effect of oligomycin, rotenone, glucose-free physiological saline, or 15% ethanol (EtOH) on cell viability using propidium iodide staining as a marker of plasma membrane permeability and cell death (*n* = 5 for each). Significance markers indicate *P* < .05 using: panel (C), ordinary one-way ANOVA with Dunnett’s test for multiple comparisons; or panel (D), repeated measures one-way ANOVA with Tukey’s test for multiple comparisons (* vs. baseline; # vs. PE; § vs. PE/ACh; † vs. PE/ACh/oligomycin).

A pharmacological concern in these experiments was that oligomycin, a potent inhibitor of mitochondrial ATP production (Figure 1F), may have interfered with other mitochondrial signaling pathways. For example, oligomycin may have increased the production of ROS. To address this issue, we used the fluorescent indicator, dihydroethidium, to image superoxide levels in freshly isolated ECs sheets (Figure 2A–C). The mitochondrial uncoupler, CCCP (5 µM), and rotenone each caused an increase in dihydroethidium fluorescence, indicative of a transient increase in ROS production. CCCP-evoked increases in ROS production were inhibited by global (TEMPO, 100 µM) or mitochondrially targeted (mitoTEMPOL, 50 µM) ROS scavenging with superoxide dismutase mimetics. However, oligomycin failed to increase endothelial ROS levels. Nonetheless, we examined if ROS scavenging could prevent the effect of oligomycin on ACh-evoked vasodilation. The global and mitochondrially targeted ROS scavengers each failed to prevent the reversal of ACh-evoked vasodilation by oligomycin (Figure 2D). These results confirm that oligomycin does not suppress EC function by increasing ROS.

We also used propidium iodide cell viability staining to examine if oligomycin promoted programmed cell death, as reported in some,^33^ but not all cells.^32^,^34^ A 60-minute exposure to either oligomycin, or rotenone, or glucose-free physiological saline had no effect on cell membrane permeability or apoptosis in native ECs (Figure 2E). In contrast, the majority of ECs exhibited nuclear propidium iodide staining following exposure to 15% ethanol. These observations rule out cell death as a cause of endothelial dysfunction following depletion of EC mitochondrial ATP.

Together, our data suggests that mitochondrial generation of ATP is critical for endothelial nitric oxide-mediated regulation of regulating vascular tone.

### Mitochondrial-Derived ATP Facilitates IP_3_-Mediated EC Calcium Signaling

We next asked how mitochondrial ATP facilitates nitric oxide- but not TRPV4-mediated vasodilation. The ACh-nitric oxide vasodilator pathway is mediated via muscarinic (M_3_ subtype^35^), G_αq_-dependent stimulation of phospholipase C and the subsequent hydrolysis of phosphatidylinositol-4,5-bisphosphate (PIP_2_) to produce IP_3_,^36^,^37^ Once liberated, IP_3_ activates IP_3_ receptors to evoke Ca^2+^ release from internal stores and Ca^2+^-dependent activation of endothelial nitric oxide synthase.^28^,^38^ In contrast, TRPV4-mediated vasodilator signaling is mediated by Ca^2+^ influx, Ca^2+^ induced Ca^2+^ release via IP_3_ receptors, and Ca^2+^-dependent initiation of endothelium-derived hyperpolarization. Because functional mitochondria are required for basal (unstimulated) EC IP_3_-mediated Ca^2+^ signaling,^39^ we hypothesized that mitochondrial ATP synthase control of nitric-oxide mediated vasodilator signaling arises via the nucleotide’s control of IP_3_-mediated Ca^2+^ signaling. We also speculated that a differential ATP requirement for Ca^2+^ release vs. Ca^2+^ influx may explain how TRPV4-mediated vasodilation persists in the absence of a functional mitochondrial ATP synthase.

**Figure 3.**
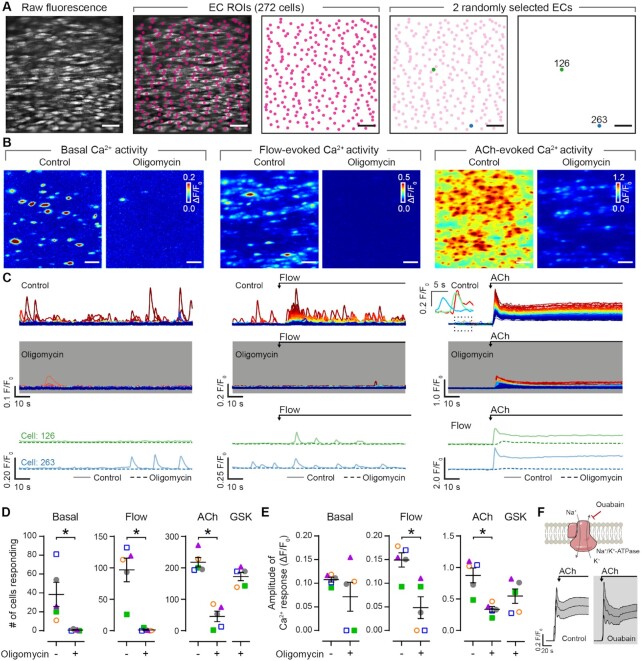
Endothelial calcium signaling requires a functional mitochondrial ATP synthase. (A) Images of population level endothelial Ca^2+^ imaging. Regions of interest (ROIs) were generated for all ECs visualized using Cal-520/AM (5 μM). (B) and (C) Ca^2+^ activity images (B) and corresponding single-cell Ca^2+^ signals (C) illustrating basal, flow-evoked, and ACh-evoked (10 μM) Ca^2+^ activity before (control) and after incubation with the mitochondrial ATPase inhibitor, oligomycin (2.4 μM). In (B), Ca^2+^ images are pseudo-colored maximum intensity projections of ΔF/F_0_ datasets (2-min recordings). Images are grouped by stimulus and the same color scale is used for both images in each set. The same *y*-axis scale is used for Ca^2+^ traces within each set, and traces are colored by the magnitude of the response to ACh. (D) and (E) Summary data showing the effect of oligomycin on endothelial Ca^2+^ activity. At the end of each experiment, Ca^2+^ responses to the TRPV4 agonist, GSK1016790A (GSK; 20 nM) were recorded. Each color-coded set of data points represents paired data from a single artery (*n* = 5, each from a different animal). The dataset in (A)–(C) is summarized in (D) and (E) as magenta triangles. The blue outlined square datapoints are shown in Videos 2–4. (F) Mean ± SEM time courses (before and after) showing the effect of the Na+/K+- ATPase inhibitor, ouabain (10 μM; *n* = 5), on ACh-evoked (10 μM) Ca^2+^ activity. Summary data are mean ± SEM; * indicates significance (*P* < .05) using paired *t*-test (basal/flow) or repeated measures one-way ANOVA with Dunnett’s test (agonist-evoked activity, vs. ACh control). Scale bars = 50 μm.

To test these hypotheses, we used high resolution, wide-field single photon imaging to probe Ca^2+^ signaling in large populations of ECs in intact arteries. Using a paired (before/after) experimental design, we examined the effect of mitochondrial ATP synthase impairment on basal, flow-evoked, and ACh-evoked EC Ca^2+^ activity, each of which are dependent on IP_3_-mediated Ca^2+^ release.^29^,^38–40^ Oligomycin inhibited each of these three endothelial Ca^2+^ signaling modalities (Figure 3A–E, Videos 2–4). As with ACh-evoked vasodilation, the effect of oligomycin was not due to Na^+^/K^+^-ATPase inhibition since the specific inhibitor of this enzyme, ouabain (10 μM), was without effect on ACh-evoked endothelial Ca^2+^ signaling (Figure 3F). Oligomycin also inhibited ACh-evoked Ca^2+^ signaling in freshly isolated EC sheets, which lack contacts with smooth muscle cells (Figure S4A–D, Video 5). Together with our observation that oligomycin did not cause vasoconstriction, these results demonstrate a direct effect of oligomycin on EC function. Moreover, the data suggest that mitochondrial ATP production modulates IP_3_-mediated Ca^2+^ activity.

**Figure 4.**
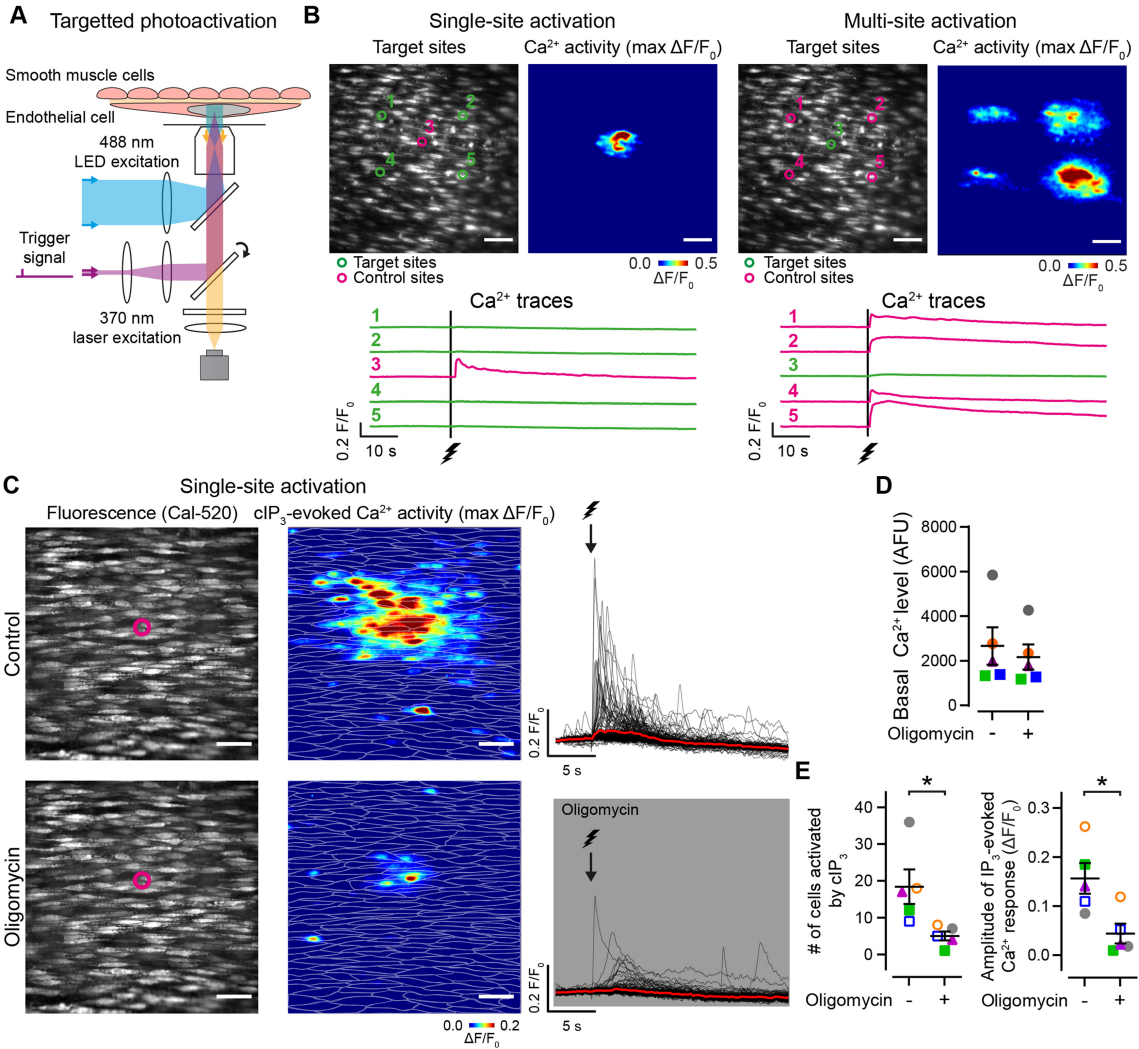
Direct activation of endothelial IP_3_ receptors requires mitochondrial ATP. (A) Schematic of single-photon microscopy with targeted EC photoactivation. (B) Example fluorescence and Ca^2+^ activity images, and corresponding Ca^2+^ traces illustrating single- and multi-site activation of EC IP_3_ receptors via photolysis of caged IP_3_ (cIP_3_). ECs were dual loaded with the Ca^2+^ indicator, Cal-520/AM and cIP_3_. Green circles denote photoactivation target regions, magenta circles indicate control (no stimulation) regions. Photolysis of caged IP_3_-evoked robust Ca^2+^ responses. (C) Representative fluorescence and Ca^2+^ activity images, and corresponding single-cell Ca^2+^ traces illustrating single-target Ca^2+^ responses before (top) and after (bottom) inhibition of the mitochondrial ATP synthase with oligomycin (2.4 μM). The bolt denotes the time the photostimulus. (D) and (E) Summary data showing the effect of oligomycin on basal Ca^2+^ levels and IP3-evoked endothelial Ca^2+^ activity. Each color-coded set of data points represents repeat measurements from a single artery (*n* = 5, each from a different animal). The dataset in (C) is summarized in (D) and (E) as magenta triangles. See Video 6. Summary data are mean ± SEM; * indicates statistical significance (*P* < .05) using paired *t*-test (*n* = 5, each from a different animal). All image scale bars = 50 μm.

To further investigate mitochondrial control of IP_3_-mediated Ca^2+^ signaling, we dual-loaded intact artery ECs with a caged form of IP_3_ and the Ca^2+^ indicator, Cal-520/AM. This strategy allowed us to activate IP_3_ receptors by photo-releasing IP_3_ using an ultraviolet laser, bypassing phospholipase C activation and IP_3_ production, whilst performing simultaneous single-photon Ca^2+^ imaging (Figure 4A and B). This experiment allowed us to distinguish between mitochondrial control of IP_3_ production and mitochondrial control of IP_3_ receptor activation. Importantly, oligomycin had no effect on basal Ca^2+^ levels (Figure 4D), and does not alter the Ca^2+^ store content in native ECs.^39^ However, Ca^2+^ activity evoked by photo-stimulated IP_3_ release was inhibited following mitochondrial ATP synthase impairment (Figure 4C and D, Video 6). Thus, mitochondrial ATP modulates IP_3_ receptor activation in native ECs.

In contrast to ACh- or directly evoked IP_3_-mediated activity, TRPV4-mediated (GSK1016790A, 20 nM) Ca^2+^ responses persisted in the presence of oligomycin (Figure 3D and E). Under normal conditions, Ca^2+^ responses evoked by TRPV4 channel activation consist of (1) a “slow,” persistent increase in Ca^2+^ arising via influx mechanisms and (2) “fast” spikes in Ca^2+^ arising via IP_3_ receptor-mediated Ca^2+^ release.^28^ We found that oligomycin had no effect on the mean amplitude of TRPV4-mediated Ca^2+^ influx, but significantly reduced the amplitude and number of TRPV4-mediated, IP_3_-dependent events (Figure S5A–D). These results suggest that mitochondrial ATP is not required for TRPV4-mediated Ca^2+^ influx, whilst providing further evidence that mitochondrial ATP fuels IP_3_-mediated Ca^2+^ signaling.

### Mitochondrial Oxidation of Pyruvate Drives Mitochondrial Control of Endothelial Function

**Figure 5.**
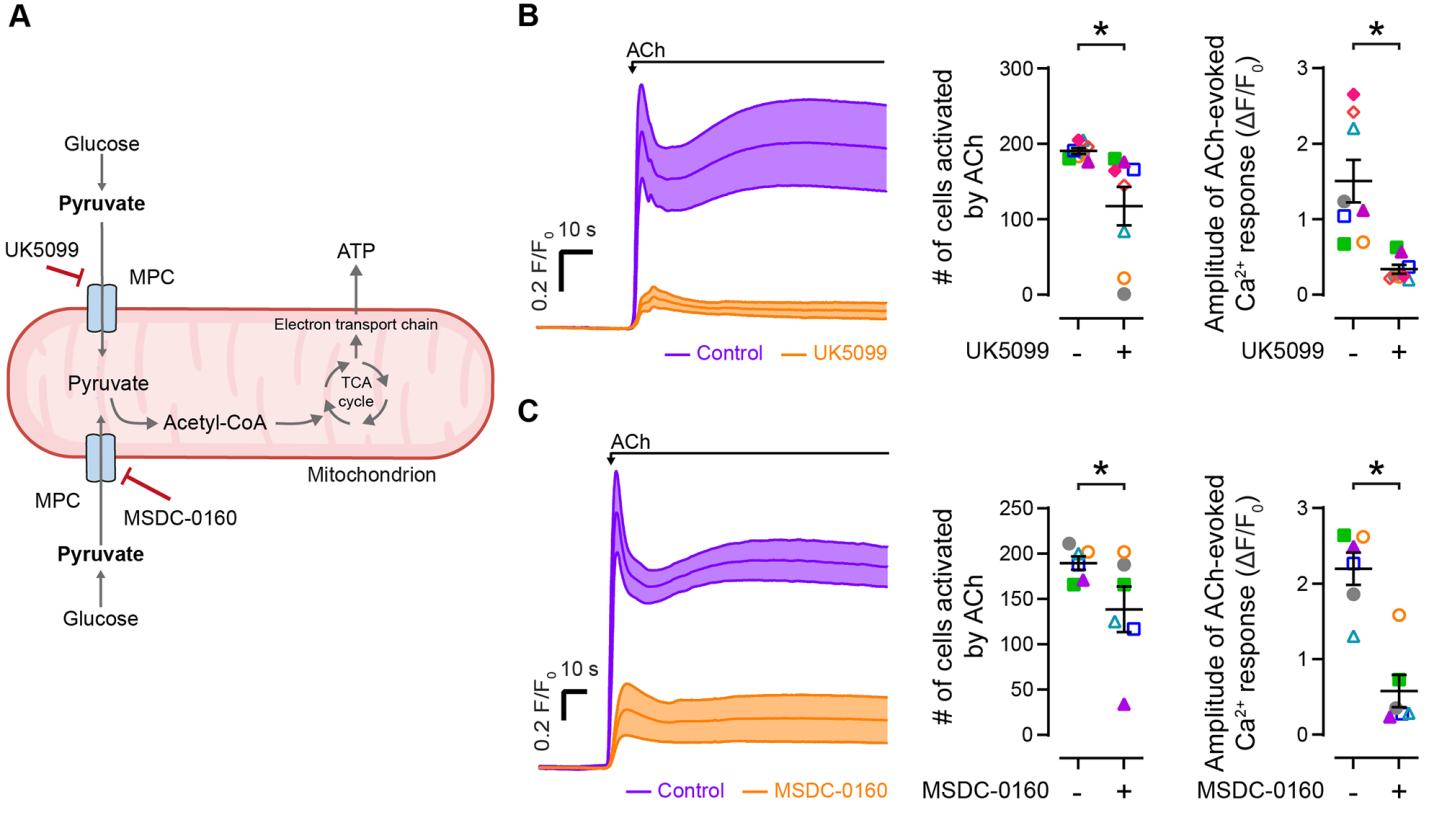
Limiting mitochondrial pyruvate transport inhibits EC calcium signaling. (A) Diagram depicting the sites of action for mitochondrial pyruvate transport inhibitors. (B) and (C) Mean ± SEM time courses (before and after), and summary data plots showing the effect of the MPC inhibitors, UK5099 (B; 50 μM; *n* = 8) and MSDC-0160 (mitoglitazone; 10 μM; *n* = 6) on ACh-evoked (10 μM) endothelial Ca^2+^ activity. Each color-coded set of data points represents repeat measurements from a single artery (each from a different animal). Summary data are mean ± SEM; * indicates statistical significance (*P* < .05) using paired *t*-test.

Since mitochondrial ATP is required for EC IP_3_-mediated Ca^2+^ signaling, we reasoned that inhibiting the transport of pyruvate—the major fuel for mitochondrial energy production—would also impair the endothelial Ca^2+^ response to ACh. Mitochondrial pyruvate metabolism requires the substrate (pyruvate) to be transported into the mitochondrial matrix by the mitochondrial pyruvate carrier (MPC).^41^ Inhibition of mitochondrial uptake of pyruvate using either UK5099 (50 μM) or MSDC-0160 (mitoglitazone; 10 μM) decreased ACh-evoked EC Ca^2+^ signaling (Figure 5A–C). The action of these inhibitors demonstrate that pyruvate oxidation is an important energy pathway in the nonproliferative (“quiescent”) ECs of the fully formed vascular wall.

### Mitochondrial ATP is a Pan-Endothelial Regulator of Vascular Function

**Figure 6.**
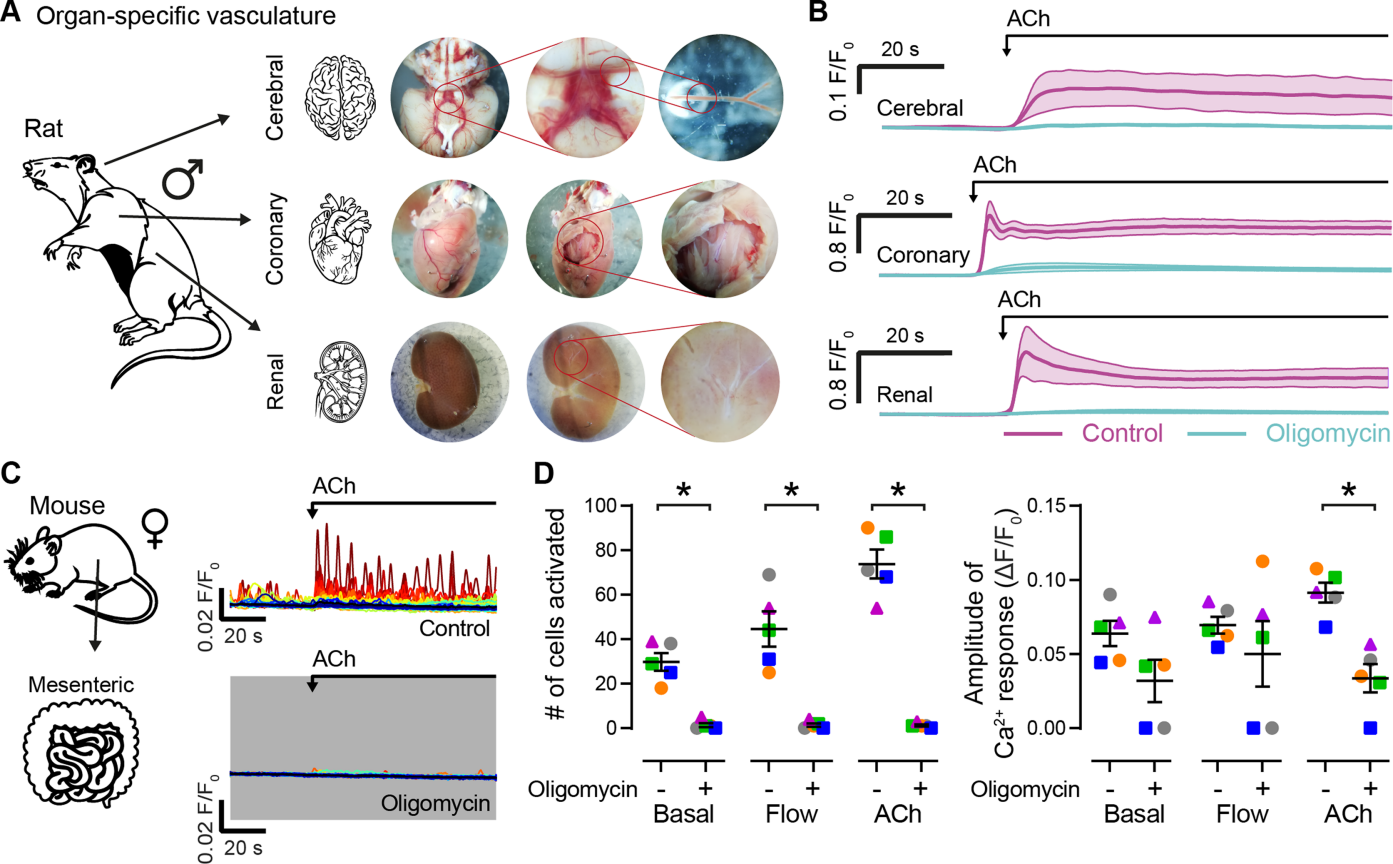
Mitochondrial ATP is a pan-endothelial regulator of calcium signaling. (A) and (B) Isolation procedure (A) and mean ± SEM Ca^2+^ signals (B; before and after) showing the effect of the mitochondrial ATPase inhibitor, oligomycin (2.4 μM), on ACh-evoked (10 μM) endothelial Ca^2+^ activity in rat cerebral (*n* = 3), coronary (*n* = 3), and renal (*n* = 3) microvessels. (C) and (D) Example single-cell Ca^2+^ traces (C; before and after), and paired summary data plots (D) showing the effect of oligomycin (2.4 μM) on basal, flow-evoked, and ACh-evoked (10 μM) EC Ca^2+^ signaling in mouse mesenteric arteries. Each color-coded set of data points represents repeat measurements from a single artery (*n* = 5, each from a different animal). The dataset in (C) is summarized in (D) as gray circles. Summary data are mean ± SEM (*n* = 5, each from a different animal); * indicates statistical significance (*P* < .05) using paired *t*-test.

As ECs exhibit a bewildering degree of heterogeneity among and within vascular beds,^42^,^43^ we asked one final question: is the control of nitric oxide vasodilator signaling by mitochondrial ATP a universal feature of EC function? To answer this, we examined muscarinic receptor-mediated endothelial Ca^2+^ activity in cerebral, coronary, and renal microvessels of the rat (Figure 6A and B). We also examined endothelial Ca^2+^ signaling in mouse mesenteric arteries (Figure 6C and D). Regardless of vascular bed examined, sex, or species, IP_3_-mediated Ca^2+^ signaling required a functional mitochondrial ATP synthase. Thus, mitochondrial ATP production appears to be a ubiquitous energy pathway in quiescent ECs of mature blood vessels.

## Discussion

### Arterial ECs Use Oxidative Phosphorylation for Blood Flow Control

**Figure 7.**
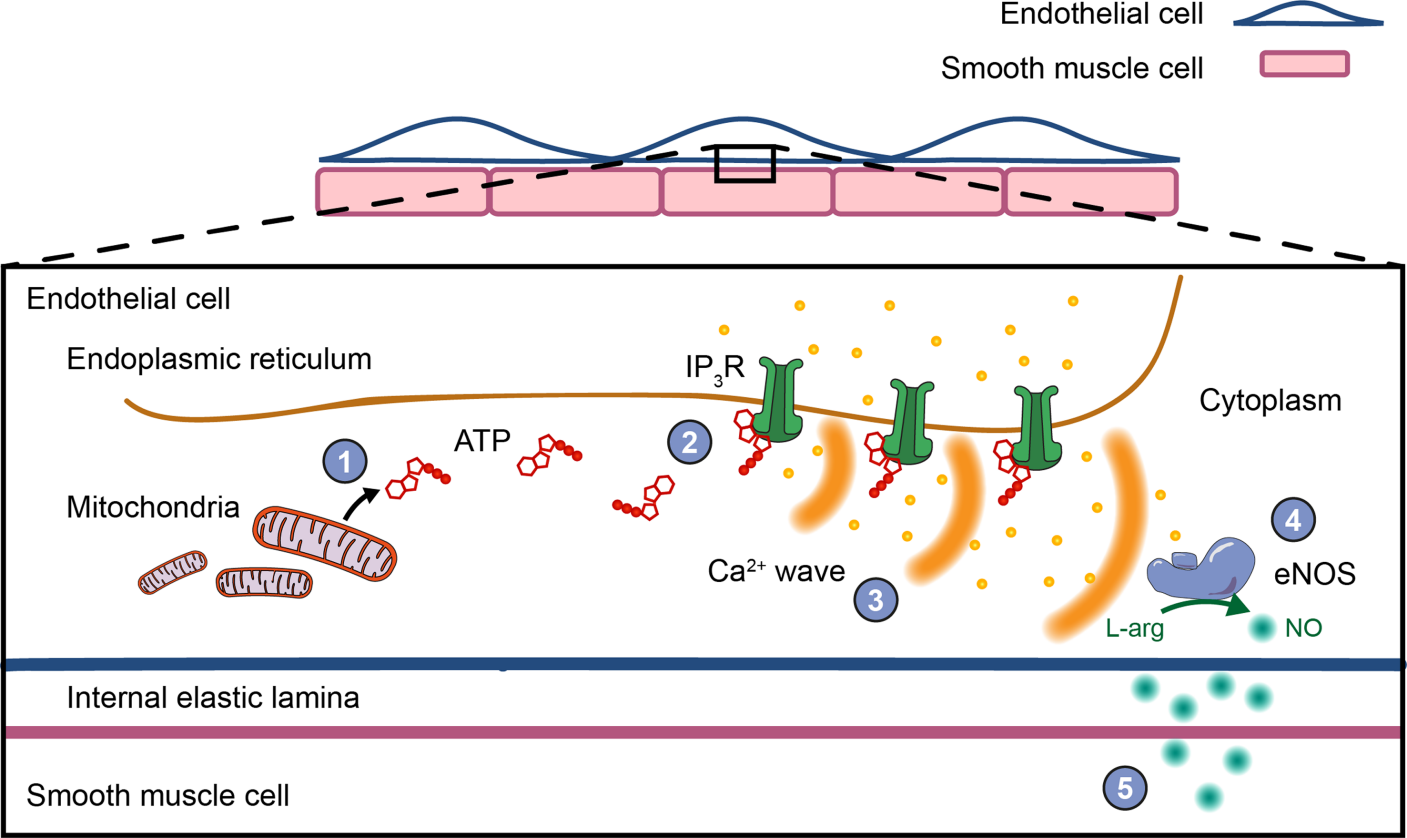
Mitochondria ATP fuels nitric oxide-mediated vasodilator signaling. Diagram illustrating the proposed pathway that implicates mitochondria as important metabolic regulators of endothelial nitric oxidemediated vasodilation. Mitochondrial-derived ATP (1) modulates IP3 receptor activity to promote Ca^2+^ release (2) and Ca^2+^ waves (3). Ca^2+^ waves stimulate nitric oxide production (4) which relaxes vascular smooth muscle.

Mammalian cells generally rely on mitochondrial respiration to fuel bioenergetic processes, but ECs are considered to be an exception to this rule. Rather than relying on mitochondrial ATP production, ECs are thought to meet approximately 85% of their energy demand via glycolysis.^6–8^,^44^ This estimation has emerged from studies examining the angiogenic potential of cultured proliferative/migratory ECs. However, the majority of ECs in perfused blood vessels are neither proliferative nor migratory (eg, less than 2% of ECs in liver and spleen have a proliferative phenotype^45^). Yet, from data derived from cultured proliferative/migratory ECs, it is assumed that “quiescent” ECs are also glycolytic and that mitochondrial-derived ATP plays little role in regulating endothelial function. In stark contrast to this assumption, we provide clear evidence that mitochondrial ATP production is crucial for the most widely known function of ECs in mature blood vessels - the control of artery diameter (Figure 7).

EC control of artery diameter is exemplified by the vascular response (vasodilation) to the classical neurotransmitter, ACh,^27^ which is released by ECs in response to fluid flow.^40^,^46^ When activated by ACh, endothelial muscarinic receptors stimulate the production of nitric oxide by endothelial nitric oxide synthase. Once produced, nitric oxide diffuses to adjacent smooth muscle cells to cause smooth muscle cell relaxation and vasodilation. In addition to nitric oxide production, EC activators may also initiate the spread of a hyperpolarizing electrical signal (endothelium-dependent hyperpolarization), that also relaxes adjacent smooth muscle cells.

In the present study, we have demonstrated that nitric oxide and endothelium-dependent hyperpolarization pathways can be independently activated in mesenteric arteries by distinct EC agonists. ACh-evoked vasodilation was abolished by nitric oxide synthase inhibitors, but persisted following the inhibition of IK and SK channels, confirming that the muscarinic receptor-mediated response is predominantly mediated by nitric oxide.^28^,^29^,^38^ The inhibitory profiles of these pharmacological agents were reversed for GSK1016790A-evoked vasodilation, demonstrating the key role of endothelium-derived hyperpolarization in the TRPV4-mediated vasodilator response.^40^,^46^ Importantly, our results show that these two vasodilator pathways are differentially modulated by mitochondrial ATP. When mitochondrial ATP production was prevented, a rapid loss of ACh-evoked endothelium-dependent and nitric oxide-mediated vasomotor control occurred—ECs failed to oppose vascular tone, and they failed to initiate vasodilation. In contrast, TRPV4-mediated vasodilation persisted following mitochondrial impairment. As smooth muscle cell contraction and endothelium-independent vasodilation were not impaired following the inhibition of mitochondrial respiration, the defect in nitric oxide signaling arises due to a direct effect on ECs and not smooth muscle cells. This conclusion is consistent with observations that endothelial mitochondria do generate ATP^5–8^,^47–52^ and that the organelle increases ATP production when challenged.^53–55^

### Mitochondrial ATP is Required for EC Calcium Release

The requirement of mitochondrial ATP synthesis for endothelial vasodilator signaling appears to be selective to the nitric oxide signaling pathway. Endothelial nitric oxide synthase is activated by intracellular Ca^2+^ signals. In unstimulated ECs, spontaneous Ca^2+^ release events via IP_3_ receptors give rise to a basal level of nitric oxide that opposes vascular tone.^29^ Mechanical and chemical stimuli each amplify this basal Ca^2+^ signaling modality, whilst also stimulating Ca^2+^ entry (eg, via Piezo1 channels^56^), to activate nitric oxide production and promote vasodilation. We observed a severe disruption of basal, flow-, agonist-, and directly evoked IP_3_-mediated Ca^2+^ signaling in respiration-deficient ECs in intact arteries and in freshly isolated sheets of ECs. Preventing the transport of pyruvate by targeting the MPC also inhibited EC Ca^2+^ signaling. These results support our interpretation that the dependence of endothelial nitric oxide-mediated vasomotor control on mitochondrial ATP arises via the nucleotides control of IP_3_-mediated Ca^2+^ release.

Further support for this conclusion is found in our observations that mitochondrial ATP is required for the IP_3_-mediated Ca^2+^ release, but not IP_3_-independent Ca^2+^ influx, component of TRPV4-mediated Ca^2+^ signals. The reason that TRPV4-mediated Ca^2+^ influx is independent of mitochondrial ATP is because ATP is not required for channel activation^30^–ATP may even suppress endothelial TRPV4 channel activity.^31^ This result is important as it provides insight into why mitochondrial ATP is not required for Ca^2+^ influx-driven, IK/SK channel-mediated vasodilation.^57^

The question arises as to how mitochondrial ATP controls Ca^2+^ release. As oligomycin does not cause a reduction in EC Ca^2+^ store content,^39^ and did not alter cytosolic Ca^2+^ levels in the present study, it is likely that ATP-mediated control of Ca^2+^ release arises via modulation of IP_3_ receptor activity. Two main mechanisms may explain how mitochondrial ATP modulates IP_3_ receptor-mediated Ca^2+^ signaling to promote endothelial vasodilator signaling. First, by binding specifically to an ATP-binding site on the IP_3_ receptor, micromolar concentrations of ATP may sensitize the channel to promote Ca^2+^ release.^58–60^ Second, the generation of IP_3_ and its precursors by phospholipase C and phosphoinositide kinases are dependent on intracellular ATP.^31^,^61–63^ Specifically in native ECs, synthesis of the IP_3_ precursor, PIP_2_, requires millimolar ATP concentrations.^31^,^64^ Thus, in the absence of sufficient cytosolic ATP, IP_3_ receptor-mediated Ca^2+^ signaling may fail. Because of our finding that Ca^2+^ responses evoked by direct activation of IP_3_ receptors require a functional mitochondrial ATP synthase, we speculate that at least part of the mitochondrial ATP requirement resides at the IP_3_ receptor. However, given that an EC PIP_2_ deficiency has been implicated in vascular dysfunction in cerebral small vessel disease,^65^ it will be important going forward to determine if a loss of mitochondrial-derived ATP also prevents IP_3_ production.

Another key question regarding IP_3_ receptor regulation is whether mitochondrial ATP also modulates IP_3_ receptor activity indirectly to control EC vasodilator function. Cytosolic Ca^2+^ levels can modulate IP_3_ receptor activity—some IP_3_ receptors are inhibited by both low and high Ca^2+^ concentrations (Ca^2+^-dependent inactivation). As such, ATP may regulate IP_3_ receptor activity by modulating the activity of the many pumps and ion channels that control cytosolic Ca^2+^ levels. But it is unlikely that this mechanism explains the present results, as endothelial cytosolic Ca^2+^ levels were not impacted by mitochondrial ATP depletion. Instead, endothelial IP_3_ receptors may be regulated by the phosphorylation of many different protein kinases that phosphorylate at IP_3_ receptors (PKA/PKG, PKB, CDK1, ERK, Fyn, and so on).^66^ Furthermore, many regulatory proteins that associate with IP_3_ receptors are also regulated by phosphorylation. Whether or not mitochondrial ATP contributes to such or other indirect regulation of IP_3_ receptor activity in ECs remains to be explored.

As well as fueling the molecular machinery responsible for intracellular Ca^2+^ signals, mitochondria regulate Ca^2+^ release/influx through the generation of ROS.^67–69^ The organelle also buffers cytosolic Ca^2+^ directly by accumulating/releasing the ion. Neither of these mitochondrial activities are likely to explain the present findings. With regards to the former, ROS are produced as a consequence of normal electron transport chain function and during mitochondrial dysfunction. For example, inhibition of mitochondrial complex V by the ATPase Inhibitory Factor 1 enhances the production of ROS,^70^ which may activate EC Ca^2+^ channels.^71^ Three observations exclude a role for ROS in the control of vascular tone by the mitochondrial ATP synthase: (1) oligomycin does not increase superoxide production in native ECs; (2) endothelial Ca^2+^ activity is insensitive to global or mitochondrial-targeted scavenging of ROS^39^; and (3) endothelial vasodilator signaling was abolished by ATP synthase inhibition in the presence of global/targeted ROS scavengers, at concentrations shown to inhibit CCCP-induced superoxide production. Mitochondrial Ca^2+^ buffering activity is equally unlikely to account for our findings. Indeed, because of the low affinity of the mitochondrial calcium uniporter for Ca^2+^ (kd ∼20–30 μM), mitochondria must be closely coupled to Ca^2+^ channels for the organelle to effectively buffer Ca^2+^ release. Ca^2+^ levels sufficient to be captured by mitochondria only occur in the immediate vicinity of active Ca^2+^ sources (distances less than a few hundred nanometers).^72^ In native ECs, mitochondria are mobile organelles that, at any moment in time, are on average a distance of ∼1 µm from Ca^2+^ release sites. At such a distance, Ca^2+^ levels are unlikely to rise more than 20 nM above resting values (∼100 nM),^73^ making the possibility that mitochondria buffer endothelial Ca^2+^ release unlikely. Furthermore, recent CRISPR/Cas9 studies question whether mitochondrial Ca^2+^ uptake is even capable of regulating IP_3_ receptor-mediated Ca^2+^ release.^74^ Finally, mitochondria may also control vasodilator signaling by promoting cell death (decreasing the number of cells participating in the response). In some cell types, mitochondrial ATP synthase inhibition promotes cell death.^33^ But in other cell types, a deficient ATP synthase protects against cell death.^32^,^34^ In our study, oligomycin did not increase cell membrane permeability or apoptosis, suggesting that a functional mitochondrial ATP synthase appears to be dispensable for EC viability, at least over the time course of the experiments. This observation is consistent with the extent to which glycolysis is required for EC functions beyond vasodilator signaling.

### The Complexity of EC Metabolism

Our data indicate that respiratory chain–linked metabolism is necessary for EC Ca^2+^ signaling and vasodilator activity in multiple vascular beds. This link between mitochondrial metabolism and endothelial function aligns with observations that polarized mitochondria are required for endothelial Ca^2+^ signaling in intact arteries^39^,^75^,^76^ and in cell cultures.^77–80^ Alongside our investigation of basal, flow- and ACh-evoked EC activity, historical findings using various mitochondrial inhibitors suggest that a requirement for mitochondrial ATP may be a general feature of endothelial vasodilator responses, irrespective of agonists, vascular bed, or species.^81–85^

While the requirement for mitochondria appears to be a common feature of EC vasomotor control, mitochondrial ATP production is generally considered to be dispensable for EC function.^86^ However, most studies have been carried out on cultured ECs. The cell culture environment significantly alters EC metabolism.^87^,^88^ Thus, it is important to understand endothelial energy production in as close to in vivo conditions as possible. In this regard, some investigators have used metabolic flux analysis to show that isolated brain capillaries (composed mostly of ECs) primarily use oxidative phosphorylation.^89^ RNA sequencing of freshly isolated single ECs is also increasingly being used, without a cell culture step, to characterize endothelial metabolic signatures.^90^,^91^ Such studies reveal a diversity of metabolic profiles (mitochondrial and glycolytic) amongst ECs of various organs.^42^,^92^ Moreover, the metabolic expression patterns exhibit a remarkable level of plasticity that correspond to distinct physiological functions. For example, angiogenic ECs appear to rely on oxidative phosphorylation and glycolysis as energy sources.^93^ Renal ECs upregulate mitochondrial ATP production to survive water deprivation,^94^ whilst cardiac ECs upregulate glycolysis in response to ischemia.^95^ Our studies used an intact tissue model and pharmacological approach to demonstrate a key role for mitochondrial metabolism in native ECs in intact arteries, and is supported by a recent transcriptomic analysis demonstrating a preference for oxidative phosphorylation in arterial ECs and a progressive switch to glycolysis along the arteriovenous axis.^96^ Thus, as the study of endothelial metabolism moves away from cell culture models, the emerging data suggest that mitochondrial energy production is more important to EC function than currently appreciated. However, ECs may be able to flexibly adjust their energy utilization according to nutrient availability. Indeed, whilst EC-specific targeting of Complex IV is embryonic lethal,^20^ and only a small percentage of pups survive induced endothelial-specific knockout of mitochondrial complex III,^22^ both complexes appear to be dispensable for adult survival. At present, there are no endothelial-specific knockdown models for any subunit of the mitochondrial ATP synthase. But our study highlights the importance of determining whether EC metabolic flexibility can overcome mitochondrial ATP synthase impairment in vivo.

### Perspective

EC function depends on an adequate supply of energy and it is well-established that most risk factors for cardiovascular disease (eg, obesity and aging) impact EC energy production and lead to vascular dysfunction.^17^ Many non-cardiovascular diseases also involve pathological blood vessel function due to EC deficits. For example, impaired EC control of blood flow is partly responsible for the vascular problems associated with diabetes, and deficient endothelial barrier function characterizes some types of neurodegeneration. Previously, others have validated the idea of targeting EC glycolysis as an antiangiogenic therapy to treat cancer. Our work highlights the idea that arterial ECs require mitochondrial respiration for the control of vascular tone and suggests that aberrant mitochondrial energy production may underlie endothelial dysfunction. As such, therapeutic intervention to promote EC mitochondrial energy production may be an effective strategy to combat vascular dysfunction in a range of diseases.

## Materials and Methods

### Animals

All animal care and experimental procedures were conducted in accordance with relevant guidelines and regulations, with ethical approval of the University of Strathclyde Local Ethical Review Panel, and were fully licensed by the UK Home Office regulations (Animals (Scientific Procedures) Act 1986, UK) under Personal and Project License authority. Animal studies are reported in compliance with the ARRIVE guidelines.^97^

Experiments were performed on arteries from male or female (8–12-wk-old) Sprague-Dawley rats and female (4–6-wk-old) C57BL6 mice from in house colonies. The animals were housed at The University of Strathclyde Biological Protection Unit. All animals were kept on a 12:12 light/dark cycle (temperature of 21°C ± 2°C, humidity of 45%–65%) under standard group housing conditions with unlimited access to water and chow (Rat and Mouse No.1 Maintenance, 801151, Special Diet Services, United Kingdom). Animals were kept in RC2F (rats) or M3 (mice) cages (North Kent Plastic, United Kingdom) with aspen wood chew sticks and hanging huts for enrichment. Animals were euthanized by cervical dislocation with secondary confirmation via decapitation in accordance with Schedule 1 of the Animals (Scientific Procedures) Act 1986.

Following euthanasia, mesenteric arcades, brains, hearts, or kidneys were removed and transferred to a physiological salt solution (PSS) of the following composition (mM): 125.0 NaCl, 5.4 KCl, 0.4 KH_2_PO_4_, 0.3 NaH_2_PO_4_, 0.4 MgSO_4_, 4.2 NaHCO_3_, 10.0 HEPES, 10.0 glucose, 2.0 sodium pyruvate, 0.5 MgCl_2_, 1.8 CaCl_2_ (adjusted to pH 7.4 with NaOH).

### Chemicals

Cal-520/AM (ab171868) and mitoTEMPO (ab144644) were purchased from Abcam (United Kingdom). Caged IP_3_ (cag-iso-2–145–100) was purchased from SiChem (Germany). ACh (A6625), apamin (A1289), dihydroethidium (D7008), GSK1016790A (G0798), oligomycin (O4876), ouabain (O3125), L-NA (N5501), L-NAME (N5751), MSDC-0160 (mitoglitazone, SML1884), PE (P6126), propidium iodide (P4864), rotenone (R8875), SNP (S0501), TEMPOL (4-HYDROXY-TEMPO; 176141), TRAM-34 (T6700), and UK5099 (PZ0160) were purchased from Sigma-Aldrich (United States). Pluronic F127 (P3000MP) was purchased from ThermoFisher (United Kingdom). Collagenase (Type 2; LS004176) was purchased from Worthington (United States). All other chemicals were obtained from Sigma-Aldrich.

### Isolated Artery Preparation

Either third/fourth order mesenteric arteries, first order posterior cerebral arteries, the septal coronary artery, or interlobular renal arteries were used for measurement of vascular reactivity or EC Ca^2+^ signaling. All arteries were rapidly dissected, cleaned of fat and adherent tissue and used immediately. Arteries were opened longitudinally, stretched to their in vivo length, and mounted on a Sylgard block insert of a custom perfusion chamber using 50 µm diameter tungsten pins. The endothelium was preferentially loaded with the membrane-permeant calcium indicator, Cal-520/AM (5 μM; in DMSO with 0.02% Pluronic F-127), at 37°C for 30 min. In a subset of experiments, ECs were also loaded with a membrane-permeant, photolabile form of IP_3_ (caged IP_3_, cIP_3;_ 1 μM). In these experiments, cIP_3_ was included in the Ca^2+^-indicator solution.

### Isolated EC Preparation

Third and fourth order mesenteric artery segments were enzymatically digested to obtain freshly isolated sheets of ECs (as described previously^39^,^98^). In brief, vessels were cut open to expose the endothelium, cut into small strips, and incubated in collagenase (Type 2, 2 mg mL^−1^) for 22 min at 37°C. The supernatant was then removed and the artery strips were gently washed three times in fresh PSS. EC sheets were then dispersed using a wide-bored, fire-polished glass pipette. ECs sheets were added to a glass-bottomed imaging chamber, for fluorescence imaging, or 8-well chamber slides (μ-slides; Ibidi, Germany), for immunocytochemistry. For Ca^2+^ imaging experiments, ECs were loaded with the membrane-permeant calcium indicator, Cal-520/AM (5 μM; in DMSO with 0.02% Pluronic F-127), at 37°C for 30 min. In ROS imaging experiments, ECs were loaded with the superoxide indicator, dihydroethidium (4 μM) at room temperature for 10 min. In cell viability experiments, we examined nuclear propidium iodide staining. In these experiments, propidium iodide (150 nM) was present in the PSS throughout the experiment.

### Immunocytochemistry

Freshly isolated EC sheets were allowed to adhere for 1 h prior to immunolabeling. Cells were fixed in 4% paraformaldehyde (Agar Scientific, United Kingdom) in phosphate buffered saline (PBS) for 20 min at room temperature. Cells were then washed three times in glycine solution (0.1 M), three times in PBS, and then permeabilized with Triton-X100 (0.2% in PBS) for 30 min. Cells were again washed three times in PBS, three times in an antibody wash solution (150 mM NaCl, 15 mM Na_3_C_6_H_5_O_7_, 0.05% Triton‐X100 in MillQ H_2_O), and incubated for 1 h with blocking solution (5% donkey serum in antibody wash solution) at room temperature. All individual wash steps were 5 min in duration. Cells were then incubated overnight at 4°C with goat antimouse platelet EC adhesion molecule (CD31/PECAM-1) primary antibody (R&D Systems catalog number AF3628) diluted in antibody buffer (1:1000; 150 mM NaCl, 15 mM Na_3_C_6_H_5_O_7_, 2% donkey serum, 1% BSA, 0.05% Triton X‐100, 0.02% sodium azide in MilliQ H_2_O). Following primary antibody incubation, cells were washed three times in antibody wash solution, and incubated for 1 h at room temperature with a fluorescent secondary antibody conjugated with Alexa Fluor 488 (donkey anti‐goat, 1:1000; A-11055) in antibody buffer. Cells were then washed three times in antibody wash solution, incubated with the nuclear stain, 4′,6-diamidino-2-phenylindole (DAPI; 4 nM), for 5 min, and finally washed three more times in PBS prior to imaging. Fluorescence images of mesenteric artery EC sheets were acquired using an inverted fluorescence microscope (TE-300; Nikon, Japan) equipped with a 100x, 1.4 numerical aperture oil immersion objective, 400/460 LED illumination (CoolLED, United Kingdom), and an iXon 888 (Andor, United Kingdom) electron multiplying CCD camera. Images were acquired using µManager software.^99^

### Assessment of EC ATP Production

Endothelial ATP production was assessed using an ATP-based Cell Titer-Glo Luminescent Cell Viability Kit (G7570; Promega, United States). In these experiments, we used a paired experimental design to assess the effect of oligomycin on ATP production in intact arteries and in freshly isolated sheets of ECs. In experiments using intact arteries, four equally sized segments of a single mesenteric microvessel were placed in separate wells of a clear-bottomed 96-well plate containing physiological saline. Two of the wells were treated with oligomycin for 40 min. In isolated cell experiments, four mesenteric arteries were pooled for each cell isolation to ensure an adequate signal in each experiment. After collagenase incubation (as above), EC sheets were dispersed into 700 µL of physiological saline. A volume of 100 µL of the cell suspension was then plated into each of six wells of a clear-bottomed 96-well plate, and left to adhere for 1 h at 37°C. Three of the wells were treated with oligomycin for 40-min. Following incubation with oligomycin, 100  µL of the Cell Titer-Glo reagent was added to each well, rocked for 2 min to induce cell lysis, and incubated for 10 min in the dark at room temperature to stabilize the luminescent signal. The ATP contents were recorded as luminescent signal, using a Hidex Sense microplate reader (Hidex, Finland). Each plate contained known ATP standards to generate a standard curve relating ATP concentrations to standard plate counts, which was used to estimate cellular ATP concentrations.

### Assessment of Vascular Reactivity

Vascular reactivity was assessed in isolated mesenteric arteries mounted en face.^28^,^29^,^38^ Arteries were visualized at 5 Hz using an inverted fluorescence microscope (TE2000; Nikon, Japan) equipped with a 20x, 0.75 numerical aperture objective, 460 nm LED illumination (CoolLED, United Kingdom), and an iXon 888 (Andor, United Kingdom) electron multiplying CCD camera. The resulting 666 µm × 666 µm field of view allowed quantification of vascular reactivity in opened arteries using VasoTracker edge-detection algorithms.^100^ All arteries used for experimentation had a luminal diameter ∼150 µm, and were perfused with PSS (37°C) at a rate of 1.5 mL min^−1^. PE-induced constriction was expressed as the percentage reduction from resting diameter. ACh-evoked vasodilation was expressed as a percentage of maximal relaxation (constricted diameter to resting diameter).

Arteries were partially constricted with PE added to the perfusate (to ∼80% of resting diameter ie, 20% constriction; ∼2 μM PE). This level of constriction enables the vessels to either dilate or constrict further under experimental pharmacological studies.^28^ Endothelial function was then assessed by the vasodilator response to ACh (10 μM). Under these control conditions, all arteries constricted to PE, exhibited >80% relaxation to ACh, and were included in subsequent analysis. Following washout, vasoconstrictor/vasodilator responses were examined once more. Underlying mechanisms of ACh-evoked vasodilation were then examined by adding additional pharmacological agents (as described in the text) to the perfusing bath solution. These experiments allowed us to test whether the drugs were able to reverse ACh-evoked vasodilation. In a subset of experiments, arteries were incubated with pharmacological agents before the second test of vascular reactivity. These experiments allowed the effects of the drugs on PE-evoked constriction and ACh-evoked vasodilation to be examined. In an additional series of experiments, ACh-evoked vasodilation was assessed before and after removal of glucose from the perfusate (equimolar substitution with mannitol). After glucose removal, the concentration of PE was increased (from 2 μM to an average of 15 μM) obtain an equivalent contraction. If ACh was impaired or absent after pharmacological intervention, SNP (100 μM) was added to the perfusate at the end of each experiment to confirm endothelium-independent vasodilation.

### EC Calcium Imaging

Intact mesenteric, cerebral, coronary, and renal artery EC Ca^2+^ activity was recorded at 10 Hz using the same microscope system used for assessment of vascular reactivity, but using a 40x, 1.3 numerical aperture oil immersion objective. The resulting 333 µm × 333 µm field of view was used to visualize large EC networks (∼250 cells). A paired experimental approach was used to examine basal (unstimulated/spontaneous), flow-evoked, and ACh-evoked Ca^2+^ activity before and after treatment with various pharmacological agents (40-min incubation), as described in the text. Basal activity (2 min) was examined in the absence of flow or pharmacological agents. Flow-evoked activity was examined during and after the initiation of flow at a rate of 1.5 mL min^−1^ (2-min recording; 30 s baseline). ACh-evoked activity was examined by adding the drug to the perfusate (2-min recording; 30 s baseline). In a separate series of experiments, TRPV4-mediated intact artery endothelial Ca^2+^ responses were evoked by the specific TRPV4 channel agonist, GSK1016790A. In another series of experiments, we examined Ca^2+^ responses evoked by the photolysis of caged IP_3_, using a computer-controlled tripled neodymium: yttrium aluminum garnet (Nd: Yag; wavelength 355 nm) laser (Rapp Optoelektronic, Germany) attached directly to the TE2000 microscope system.^40^ In yet another series of experiments, we examined ACh-evoked activity in isolated sheets of mesenteric artery ECs. Again, ACh was added to the perfusate and a paired experimental design was employed to examine the effects of pharmacological manipulation on endothelial Ca^2+^ signaling. All images were acquired using µManager software.^99^

### Analysis of Calcium Activity

Single-cell endothelial Ca^2+^ activity was assessed as previously described.^38^,^40^ In brief, we used automated algorithms to extract fluorescence intensity as a function of time from circular regions of interest (6.5 µm diameter) centered on each cell in our images. Fluorescence signals were then smoothed using a Savitzsly-Golay (21 point, third-order) filter, and expressed as fractional changes in fluorescence (F/F_0_) from baseline (F_0_). The baseline was automatically determined by averaging the fluorescence intensity of the 100-frame portion of each trace that exhibited the least noise. We then calculated the discrete derivative (d(F/F_0_)/dt) of each Ca^2+^ signal, and used a peak-detection algorithm to identify increases in fluorescence intensity that rose at least 10 SD above baseline noise. Endothelial Ca^2+^ activity was quantified using the number (or percentage) of cells exhibiting spiking activity, and the amplitude of these Ca^2+^ spikes (ΔF/F_0_).

### Analysis of ROS Production

Superoxide levels (dihydroethidium fluorescence) in isolated EC sheets were recorded at 0.5 Hz using the microscope system describe above for Ca^2+^ imaging. In these experiments, we used an unpaired approach to assess the effect of ROS scavengers on superoxide production evoked by mitochondrial inhibitors. The endothelium was imaged whilst the various mitochondrial inhibitors were introduced via the perfusate. In separate experiments, ECs were first incubated with ROS scavengers for 40 min, and then superoxide levels were monitored whilst mitochondrial inhibitors were introduced via the perfusate.

### Analysis of EC Viability

EC viability was assessed in intact arteries using the membrane impermeant nuclear marker, propidium iodide. In these experiments, we used a longitudinal approach to assess the effect of mitochondrial inhibitors on cell membrane integrity and cell death. Intact arteries were incubated with inhibitors and propidium iodide staining was assessed at 10-min intervals for 60 min. We also examined the effects of a 60-min period of glucose removal or a 10-min exposure to 15% ethanol on propidium iodide staining.

### Statistical Analysis

For all experiments, the reported *n* represents the number of biological replicates (number of animals). Sample sizes were chosen to achieve a minimum of 80% power at α = 0.05. Summary data are presented in text as mean ± standard error of the mean (SEM), and graphically as mean ± SEM (time–course data) or individual data points with the mean ± SEM indicated. Paired data in plots are indicated by the shape and color of the plotted points. Data were analyzed using paired *t-*tests, independent 2-sample *t-*tests (with Welch’s correction as appropriate), repeated measures one-way ANOVA with Tukey’s or Dunnett’s test for multiple comparisons, as appropriate and as indicated in the respective figure legend. All statistical tests were two-sided. A *P*-value of < .05 was considered statistically significant.

## Supplementary Material

zqac063_Supplemental_Files

## Data Availability

The data underlying this article will be shared on reasonable request to the corresponding author(s).
