# Supplementary material for: Mitochondrial ATP Production is Required for Endothelial Cell Control of Vascular Tone
Source: Function (Oxf). 2022 Dec 9;4(2):zqac063. doi: 10.1093/function/zqac063 (PMC9909368; doi:10.1093/function/zqac063)
Supplement: zqac063_Supplemental_Files [file zqac063_supplemental_files.zip › MitoATP_Paper_20221205_SI.docx]

Supplementary Information

Mitochondrial ATP production is required for endothelial cell control of vascular tone

Calum Wilson*_,_ Matthew D. Lee, Charlotte Buckley, Xun Zhang, & John G. McCarron*

Strathclyde Institute of Pharmacy and Biomedical Sciences, University of Strathclyde, 161 Cathedral Street, Glasgow G4 0RE, UK.

Short title: Mitochondria fuel endothelial cell control of vascular tone

Keywords: endothelial cell, mitochondria, ATP, vasodilation, blood flow

**Author correspondence:** Calum Wilson (c.wilson@strath.ac.uk) or John G McCarron (john.mccarron@strath.ac.uk), Strathclyde Institute of Pharmacy and Biomedical Sciences, University of Strathclyde, 161 Cathedral Street, Glasgow, G4 0RE, UK.


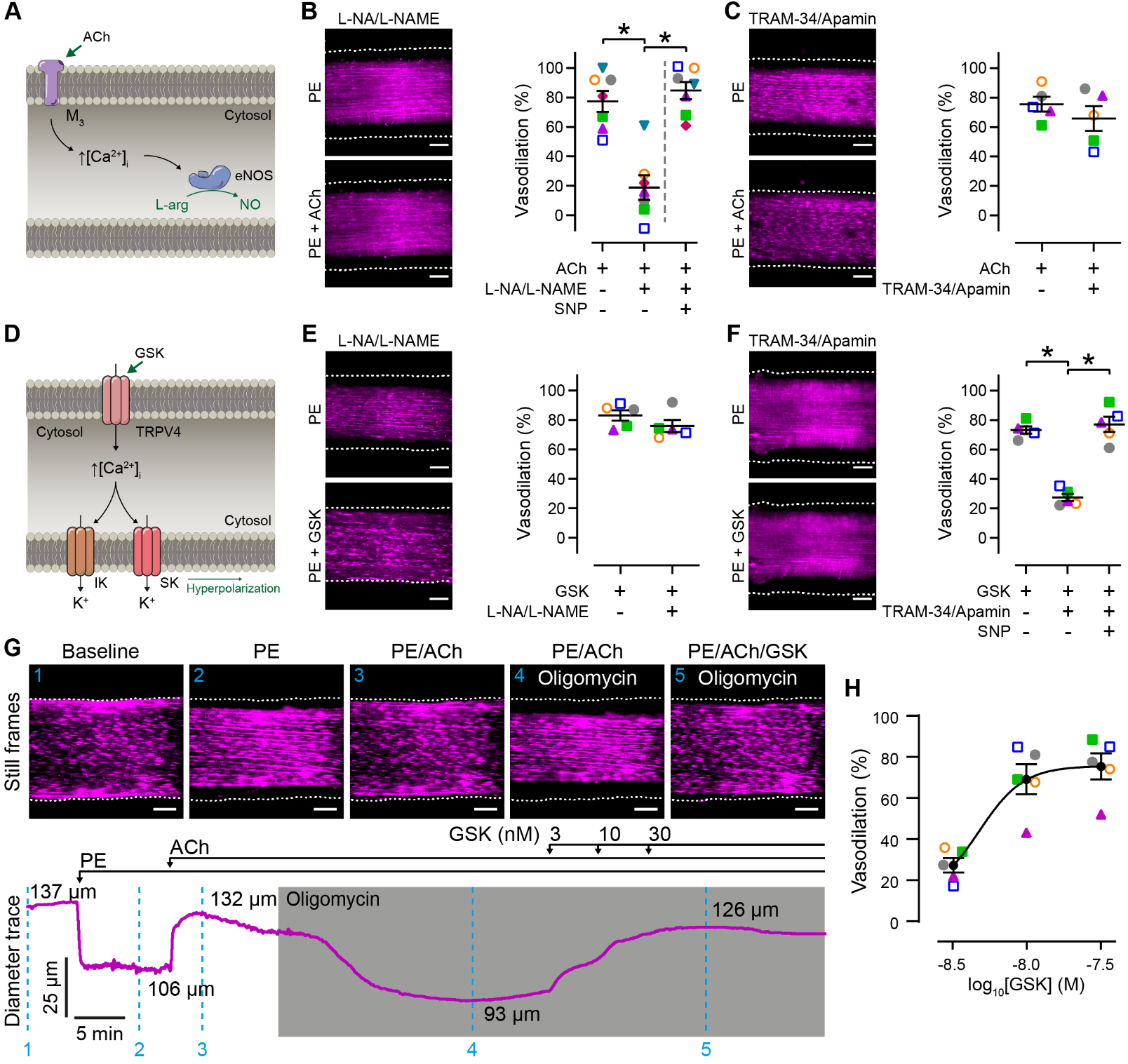


Supplementary Figure 1 – Endothelial-derived hyperpolarization-mediated vasodilation is preserved after inhibition of the mitochondrial ATP synthase.

(A-F) Acetylcholine (ACh) and TRPV4-evoked vasodilation arise via distinct signaling mechanisms. Signaling diagram and example/summary data plot showing the effect of endothelial nitric oxide synthase inhibition or Ca^2+^-activated K^+^ channels blockade on ACh-evoked (10 µM; A-C) or TRPV4-mediated (GSK1016790A, GSK; 20 nM; D-F) changes in vessel tone. (G-H) Still frame images and corresponding diameter trace (G), and summary data (H) showing the concentration dependence of TRPV4-mediated vasodilation following pharmacological inhibition of the mitochondrial ATP synthase using oligomycin (2.4 µM). Vasodilation data expressed as percentage of maximal relaxation (constricted diameter to resting diameter). Each colored data point represents measurements from a single artery (each from a different animal, n ≥ 5 for each experiment). Summary data are mean ± SEM; * indicates statistical significance (p < 0.05) assessed using paired t-test (C & E) or repeated measures one-way ANOVA with Dunnett’s test (B & F). Scale bars = 100 µm.


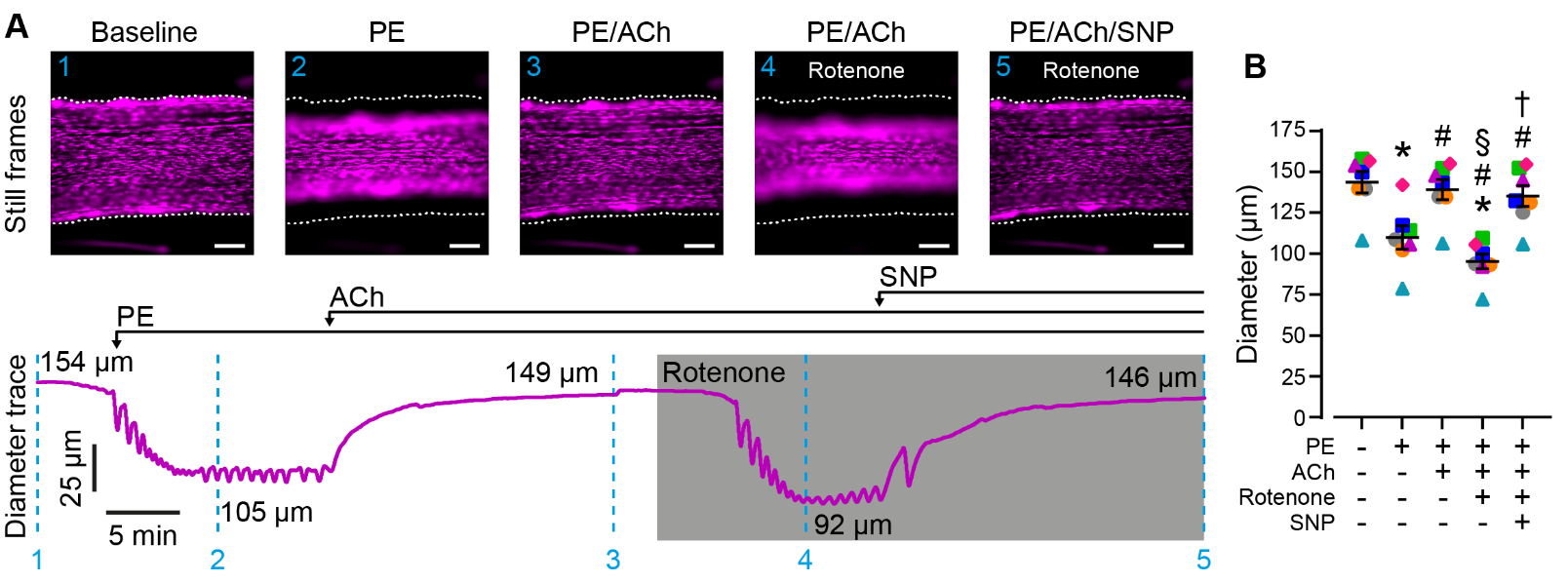


**Supplementary Figure** **2 – Inhibiting mitochondrial complex I reverses endothelium-dependent vasodilation.**

(A-B) Still frame images, diameter traces, and summary data showing the effect of mitochondrial complex I inhibition (using rotenone; 500 nM) on acetylcholine-evoked (ACh, 10 µM) vasodilation in phenylephrine (PE) constricted arteries. Sodium nitroprusside (SNP, 100 µM) was used to test endothelium-independent vasodilation. Summary data (mean ± SEM overlaid) are colored to highlight measurements from a single artery (n = 7, each from a different animal). Data in A are shown in corresponding summary data as magenta triangles. Significance markers indicate p < 0.05 using repeated measures one-way ANOVA with Tukey’s test for multiple comparisons (* vs baseline; # vs PE; § vs PE/ACh; † vs PE/ACh/oligomycin). Image scale bars = 100 µm.

**
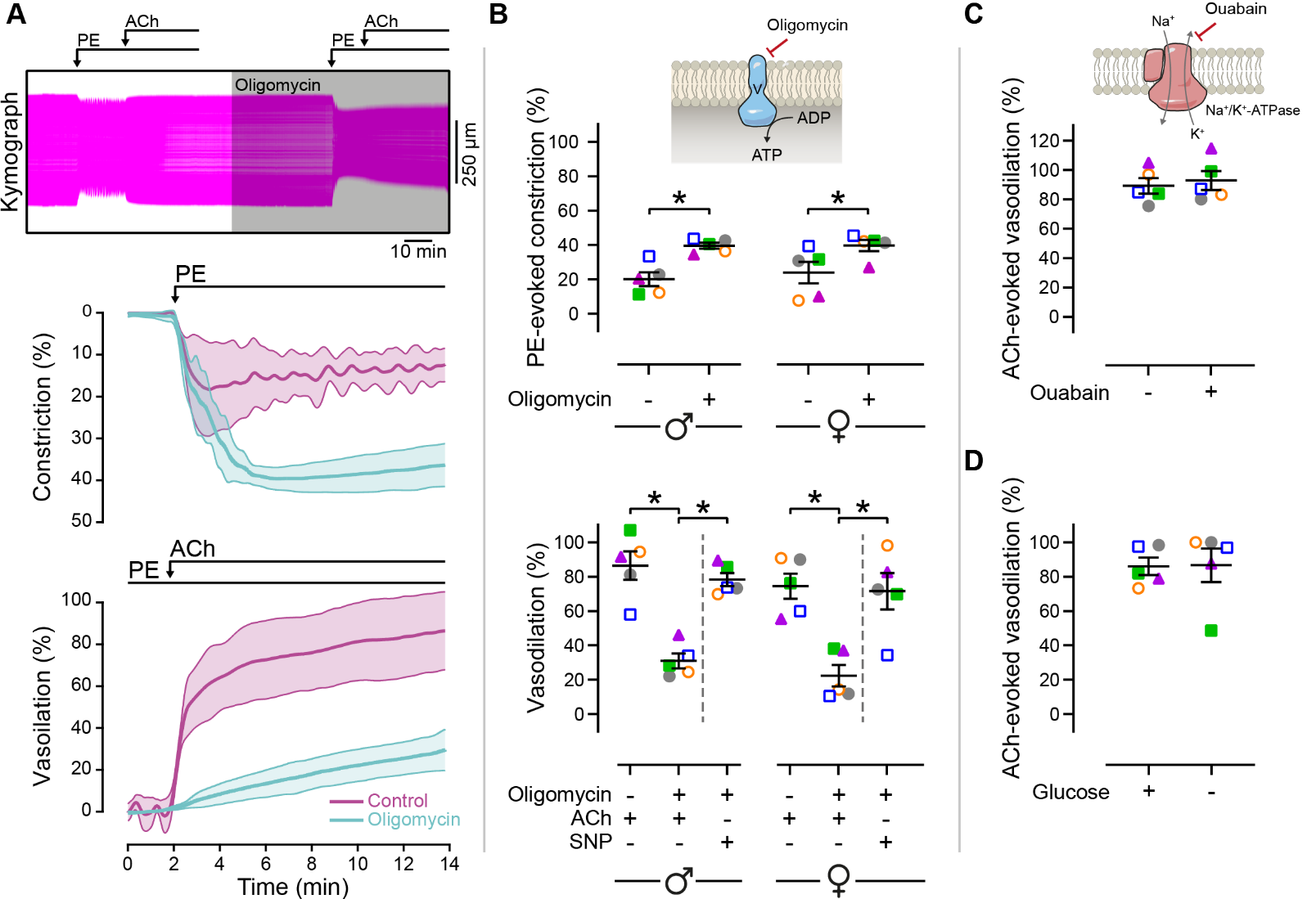
**

**Supplementary Figure 3 – Oxidative phosphorylation is required for endothelial modulation of vascular tone.** (A) Kymograph and mean ± SEM time courses of phenylephrine (PE)-evoked constriction and acetylcholine-evoked (ACh, 10 µM) dilation before and after incubation with oligomycin (n = 5; 2.4 µM). Constriction data expressed as % of initial diameter, vasodilation data expressed as percentage of maximal relaxation (constricted diameter to resting diameter). (B-D). Paired summary data plots (mean ± SEM overlaid) showing the effect of oligomycin (B), ouabain (10 µM; C), or glucose removal (D) on artery constriction/vasodilation. Individual data points are color-coded to indicate measurements from a single artery (each from a different animal, n = 5 in each experiment). * indicates statistical significance (p < 0.05) using paired t-test or repeated measures one-way ANOVA with Dunnett’s test for multiple comparisons.


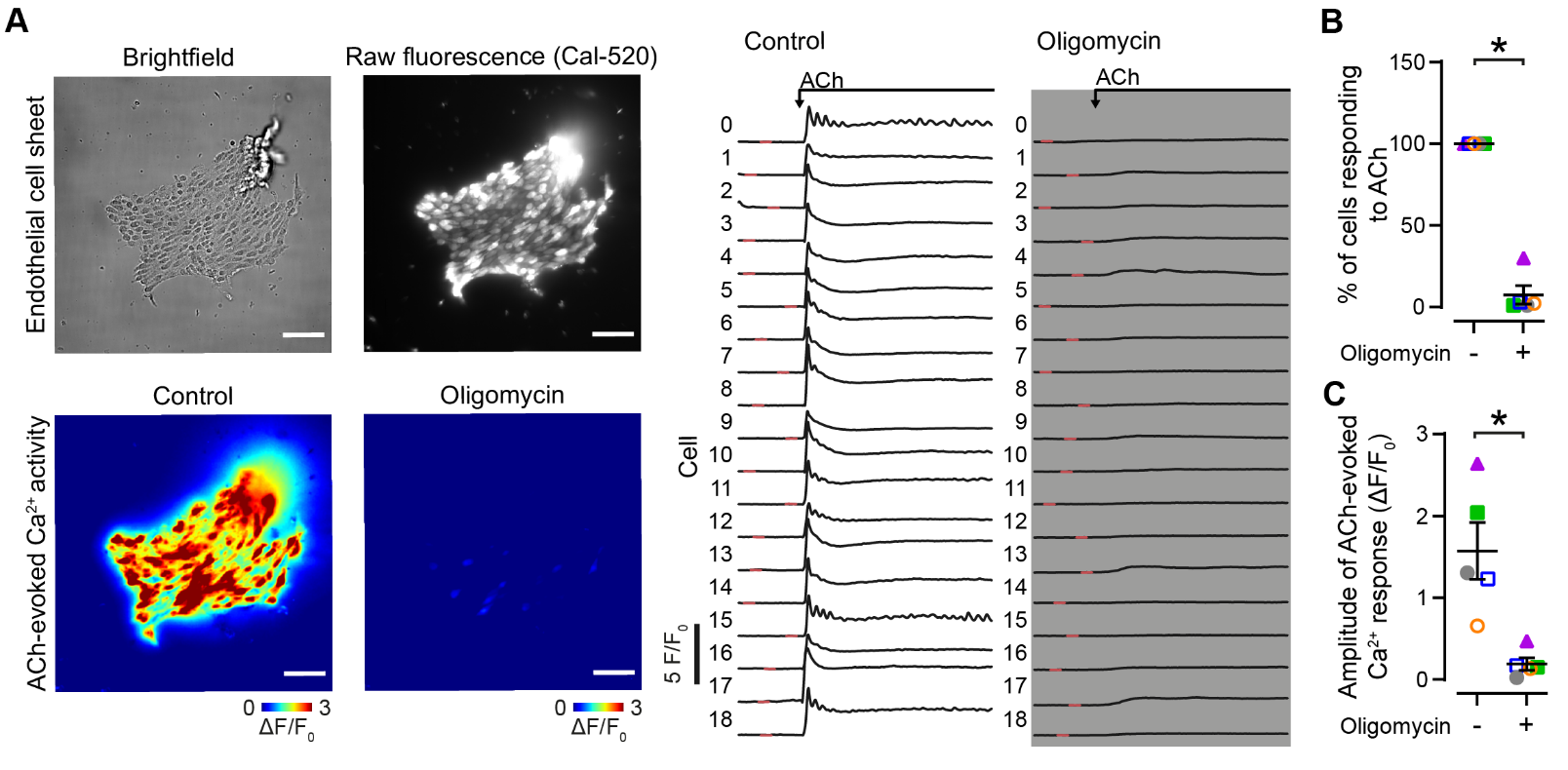


**Supplementary Figure 4 – Mitochondrial ATP is essential for calcium signaling in isolated endothelial cells.**

(A) Representative brightfield, fluorescence and Ca^2+^ activity images, and corresponding single cell traces illustrating acetylcholine-evoked (ACh; 10 µM) Ca^2+^ activity in isolated endothelial cell sheets before and after inhibition of the mitochondrial ATP synthase using oligomycin (2.4 µM). Ca^2+^ images are pseudo colored maximum intensity projections of ΔF/F_0_ datasets (2-minute recordings). (B-C) Summary data showing the effect of oligomycin on ACh-evoked Ca^2+^ activity in isolated endothelial cell sheets. Each color-coded set of data points represents repeat measurements from a single artery (n = 5, each from a different animal). The dataset in A is summarized in B-C as magenta triangles and shown in Video 5. Summary data are mean ± SEM; * indicates statistical significance (p < 0.05) using paired t-test. For presentation purposes, the data in C is shown as the percentage of cells responding to ACh, statistical tests were performed on the original cell counts. Scale bars = 50 µm (25 µm, inset in A).

**
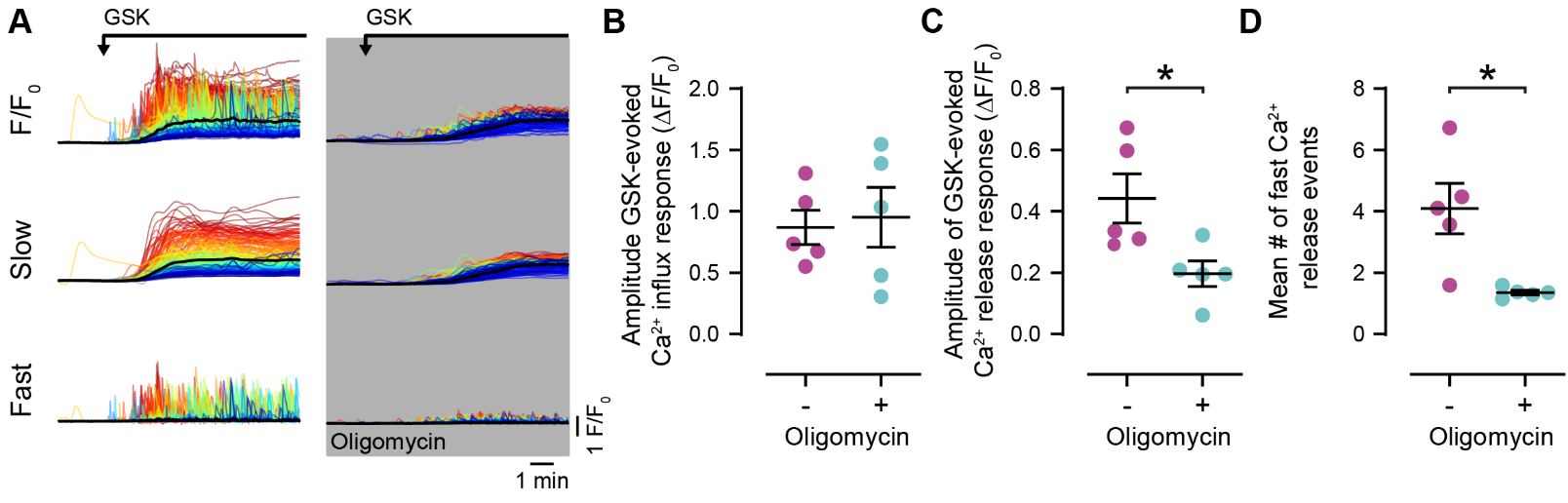
**

**Supplementary Figure 5 – Mitochondrial ATP is required for fast Ca^2+^ release events but not slow global Ca^2+^ elevations.**

(A) Single-cell signals illustrating the effect of oligomycin (2.4 µM) on mesenteric artery endothelial Ca^2+^ responses to TRPV4 activation with GSK1016790A (GSK, 20 nM). F/F_0_ signals (top) were demultiplexed into slow (middle) and fast (bottom) components which reflect Ca^2+^ influx and Ca^2+^ release, respectively. (B-D) Summary data showing the effect of oligomycin on the amplitude of the slow (B), fast (C) components of GSK-evoked Ca^2+^ responses, and the mean number of fast Ca^2+^ events (D). * indicates statistical significance (p < 0.05) using Student’s t-test (n = 5, each from a different animal).

# Video legends

Video 1: Inhibition of the mitochondrial ATP synthase reverses acetylcholine-evoked vasodilation.

Representative videos, without (left) and with (right) VasoTracker tracking results overlaid, showing the effect of oligomycin on the vasodilation response of a third order mesenteric artery. Arteries were splayed open, pinned flat, before endothelial cells were labelled with the fluorescent indicator, Cal-520/AM (5 µM), and visualized using wide field single-photon microscopy (16X objective, NA = 0.8, ~0.69 mm^2^ field of view, 0.66 µm^2^ projected pixel size). The width of artery segments (unfolded circumference) was converted to the equivalent diameter to illustrate the time course of the mechanical response (bottom). At the plateau phase of contractions evoked by phenylephrine (PE, titrated to achieve 20% contraction), acetylcholine (ACh, 10 µM) caused relaxation of the preparations. Subsequent addition of the mitochondrial ATPase inhibitor, oligomycin (2.4 µM), reversed this relaxation and increased contraction to levels greater than that induced by PE alone. Videos show the central section (832 µm × 832 µm field of view) of an ~ 3 mm long blood vessel at pixel size = 0.66 µm^2^/pixel. Data also displayed in Figure 1.

Video 2: Spontaneous endothelial calcium signaling requires mitochondrial ATP production.

Representative videos of basal (spontaneous) endothelial Ca^2+^ activity before (left) and after (right) incubation with the mitochondrial ATPase inhibitor, oligomycin (2.4 µM). Arteries were splayed open and pinned flat before endothelial cells were preferentially labelled with the fluorescent Ca^2+^ indicator, Cal-520/AM (5 µM), and visualized using high-resolution wide field single-photon microscopy (40X objective, NA = 1.3, 333 µm × 333 µm field of view, 0.11 µm^2^ projected pixel size). Ca^2+^ imaging data is displayed as fractional change in fluorescence movies (F/F_0_). Ca^2+^ traces are shown for 5 randomly selected cells, and are illustrated as a heatmap for all 197 cells contained within the field of view. Under control conditions (left), quiescent endothelium exhibits substantial spontaneous Ca^2+^ activity. After incubation with oligomycin (right), quiescent endothelium exhibit little spontaneous Ca^2+^ activity. The dataset in the movie is shown in summarized data (Figure 3D) as blue-outlined squares.

Video 3: Flow-evoked endothelial calcium signaling requires mitochondrial ATP production.

Representative videos of flow-evoked (1.5 ml min^-1^) endothelial Ca^2+^ activity before (left) and after (right) incubation with the mitochondrial ATPase inhibitor, oligomycin (2.4 µM). Arteries were splayed open and pinned flat before endothelial cells were preferentially labelled with the fluorescent Ca^2+^ indicator, Cal-520/AM (5 µM), and visualized using high-resolution wide field single-photon microscopy (40X objective, NA = 1.3, 333 µm × 333 µm field of view, 0.11 µm^2^ projected pixel size). Ca^2+^ imaging data is displayed as fractional change in fluorescence movies (F/F_0_). Ca^2+^ traces are shown for 5 randomly selected cells, and are illustrated as a heatmap for all 197 cells contained within the field of view. Prior to oligomycin treatment (left), flow of physiological saline amplifies spontaneous endothelial Ca^2+^ activity. After incubation with oligomycin (right), the endothelium exhibits little spontaneous Ca^2+^ activity and flow is without effect. The dataset in the movie is summarized in Figure 3D, and shown as blue-outlined squares.

Video 4: Acetylcholine-evoked endothelial calcium signaling requires mitochondrial ATP production.

Representative videos of acetylcholine (ACh)-evoked (10 µM) endothelial Ca^2+^ activity before (left) and after (right) incubation with the mitochondrial ATPase inhibitor, oligomycin (2.4 µM). Arteries were splayed open and pinned flat before endothelial cells were preferentially labelled with the fluorescent Ca^2+^ indicator, Cal-520/AM (5 µM), and visualized using high-resolution wide field single-photon microscopy (40X objective, NA = 1.3, 333 µm × 333 µm field of view, 0.11 µm^2^ projected pixel size). Ca^2+^ imaging data is displayed as fractional change in fluorescence movies (F/F_0_). Ca^2+^ traces are shown for 5 randomly selected cells, and are illustrated as a heatmap for all 197 cells contained within the field of view. Prior to oligomycin treatment (left), the addition of ACh to the flowing physiological saline evokes a large, global increase in endothelial Ca^2+^ levels. After incubation with oligomycin (right), the endothelium exhibits little Ca^2+^ activity despite ongoing flow, and the response to ACh is muted. The dataset in the movie is summarized in Figure 3D, and shown as blue-outlined squares.

Video 5: The endothelial requirement for mitochondrial ATP is preserved in freshly isolated cells.

Representative videos of acetylcholine (ACh)-evoked (10 µM) Ca^2+^ activity in an isolated sheet of endothelial cells before and after incubation with the mitochondrial ATPase inhibitor, oligomycin (2.4 µM). Endothelial cells were enzymatically dissociated from mesenteric arteries and labelled with the fluorescent Ca^2+^ indicator, Cal-520/AM (5 µM), and visualized using high-resolution wide field single-photon microscopy (40X objective, NA = 1.3, 333 µm × 333 µm field of view, 0.11 µm^2^ projected pixel size). Ca^2+^ imaging data is displayed as pseudocolored fractional change in fluorescence (F/F_0_). Ca^2+^ traces were extracted automatically from all cells and traces are shown: for 5 randomly selected cells, and as a heatmap for all 210 cells contained within the visualized sheet. Prior to oligomycin treatment (left), the addition of ACh to the flowing physiological saline evokes a large, global increase in endothelial Ca^2+^ levels. After incubation with oligomycin (right), the response to ACh is muted. The dataset in the movie is summarized in Supplementary Figure 4.

Video 6: Directly-evoked IP_3_-mediated endothelial calcium signalling requires mitochondrial ATP synthesis.

Representative videos of IP_3_-evoked endothelial Ca^2+^ activity before (left) and after (right) incubation with the mitochondrial ATPase inhibitor, oligomycin (2.4 µM). Arteries were splayed open and pinned flat before endothelial cells were preferentially labelled with a fluorescent Ca^2+^ indicator (Cal-520/AM, 5 µM) and caged IP3, and visualized using high-resolution wide-field single-photon microscopy (40X objective, NA = 1.3, 333 µm × 333 µm field of view, 0.11 µm^2^ projected pixel size). Ca^2+^ imaging data is displayed as fractional changes in fluorescence movies (F/F_0_). Ca^2+^ activity was evoked by laser targetted photorelease of IP_3_ in four regions (indicated by coloured circles). Average traces for each region are shown. Oligomycin inhibits Ca^2+^ signals evoked by laser-targeted photorelease of IP_3_.
