# Supplementary figures and images for: Mitochondrial ATP Production is Required for Endothelial Cell Control of Vascular Tone
Source: Function (Oxf). 2022 Dec 9;4(2):zqac063. doi: 10.1093/function/zqac063 (PMC9909368; doi:10.1093/function/zqac063)

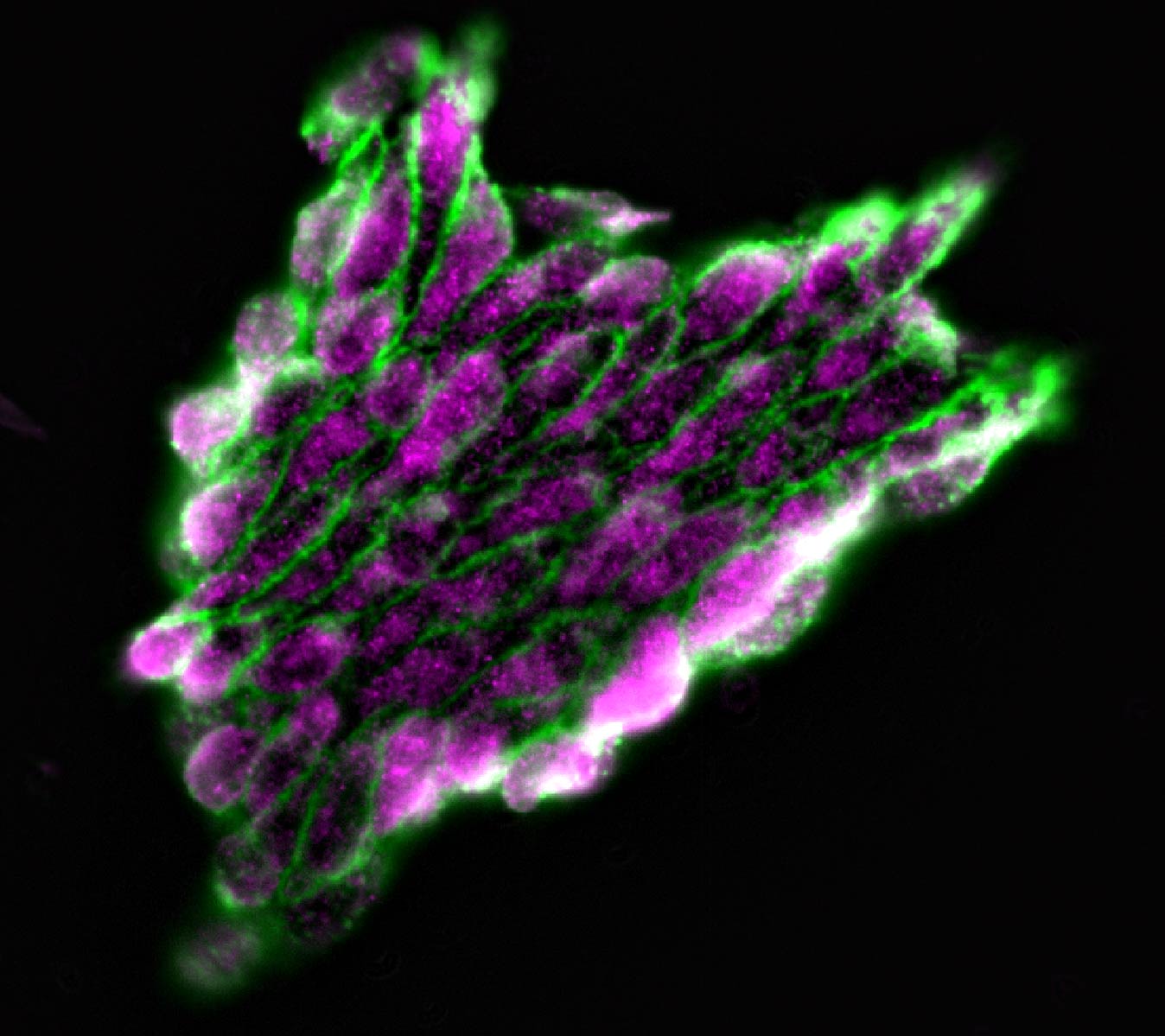

Supplement: zqac063_Supplemental_Files [file zqac063_supplemental_files.zip › Potential Cover Image.jpg]
